# Regulatory T cells in skin utilize the Cxcr4-Cxcl12 axis to promote hair follicle regeneration

**DOI:** 10.1016/j.celrep.2025.116467

**Published:** 2025-10-22

**Authors:** Jarish N. Cohen, Gayatri Kolluri, Sean Clancy, Victoire Gouirand, Courtney E. Macon, Lokesh A. Kalekar, Michael D. Rosenblum

**Affiliations:** 1 Department of Dermatology, University of California, San Francisco, San Francisco, CA, USA; 2 Department of Pathology, University of California, San Francisco, San Francisco, CA, USA; 3 Vertex Pharmaceuticals, Boston, MA, USA; 4 Lead contact

## Abstract

Regulatory T cells (Tregs) play important immunosuppressive and tissue-regenerative functions in skin. A subset of Tregs localizes to hair follicles (HFs), where they promote hair growth through activation of HF stem cells. However, the mechanisms driving Treg accumulation in HFs remain to be identified. We find that Tregs utilize Cxcr4 to accumulate in HF epithelium and that its expression is partially dependent on glucocorticoid receptor signaling. Additionally, we show that Cxcl12, the main cognate ligand of Cxcr4, is enriched in keratinocytes of the upper HF and that disruption of the Cxcr4-Cxcl12 axis results in suboptimal hair growth. Finally, we demonstrate that upper HF keratinocytes in human skin express Cxcr4 ligands in a pattern similar to that in murine skin. Collectively, these results reveal an evolutionary conserved pathway of Treg trafficking within a barrier tissue that promotes hair regeneration, which may have implications for immunotherapeutic modulation of human alopecia.

## In brief

Cohen et al. show that glucocorticoid receptor-dependent Cxcr4 expression by skin Tregs is partially required for accumulation to Cxcl12-expressing isthmic/infundibular regions of the hair follicle. The Cxcr4/Cxcl12 axis is required for optimal hair growth in mice and is an evolutionally conserved chemotactic pathway in human skin.

## Graphical Abstract

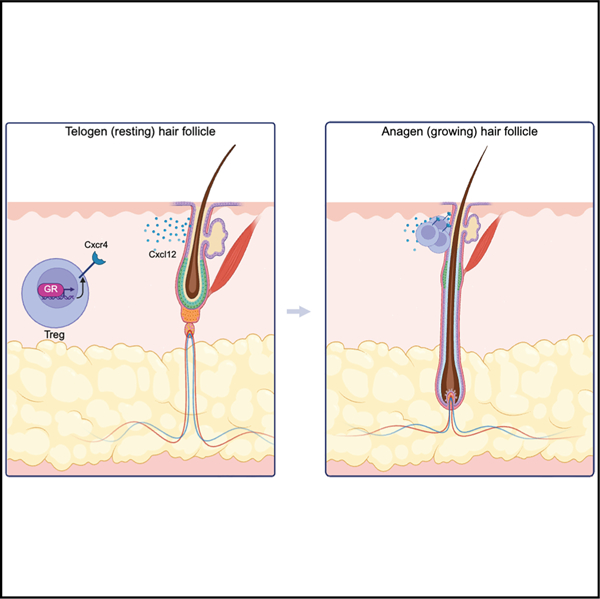

## INTRODUCTION

Regulatory T cells (Tregs) are specialized T lymphocytes that prevent systemic autoimmunity. In addition to their firmly established role in immune regulation, the “non-classical” functions of Tregs are increasingly appreciated. These include, but are not limited to, tissue repair following inflammation in muscle, lung, brain, and the gut and metabolic homeostasis in visceral fat.^1^ In skin, a subset of Tregs accumulates around hair follicle (HF) epithelium in the bulge region where HF stem cells (HFSCs) reside.^2,3^ Given their localization to this specialized microanatomic niche, Tregs have been shown to influence HFSC differentiation and migration during HF cycling and following superficial cutaneous injury.^2,4,5^ Despite advances in knowledge regarding the effector molecules Tregs utilize to promote cutaneous tissue regeneration via modulation of HFSCs, the chemotactic mechanisms that underlie Treg accumulation in the HF niche remain to be elucidated. A greater mechanistic understanding of the trafficking requirements that guide Tregs to HFs may have broad therapeutic implications for various forms of alopecia.

Lymphocytes express a variety of cell-surface molecules that permit trafficking and accumulation in various tissues, including adhesion molecules and chemokine receptors.^6^ Distinct patterns of homing molecule expression are necessary for recruitment of T cells into various secondary lymphoid organs and peripheral tissues during the steady state and following injury and/or infection. Expression of Ccr7 and CD62L have been shown to promote naive T cell trafficking to lymph nodes (LNs).^7,8^ Additionally, the integrin α4β7 and Ccr9 play a crucial roles in promoting accumulation of activated effector T cells in the gut.^9,10^ P- and E-selectin and Ccr4 have been shown to play an integral role in mediating T cell, including Treg, trafficking to skin during homeostasis and inflammation.^11–13^ Moreover, Cxcr3 expression by effector T cells promotes skin trafficking in a mouse model of alopecia areata.^14^ While much is known about the molecular mechanisms that enable lymphocytes to enter barrier tissues, the pathways that govern T cell trafficking to specific microanatomic niches within a given peripheral tissue remain largely unknown.

To better understand how skin Tregs localize to HF epithelium, we combined characterization of chemokine expression by cutaneous keratinocyte subsets with expression of cognate receptors on skin Tregs. In this manner, we found that keratinocytes in the infundibular and isthmic region of HFs are highly enriched for Cxcl12 expression. Additionally, we show that Cxcr4 expression on skin Tregs promotes their accumulation in HF epithelium. Intriguingly, expression of the glucocorticoid receptor (GR) by skin Tregs maintained Cxcr4 expression. In functional experiments, we demonstrate that Cxcr4 on skin Tregs promotes HF regrowth by promoting HFSC proliferation. Thus, the Cxcr4-Cxc12 chemotactic axis appears to be an evolutionarily conserved mechanism of *in situ* homing that promotes cutaneous tissue regeneration.

## RESULTS

### Cxcl12 is enriched in HF keratinocytes

**Figure 1.**
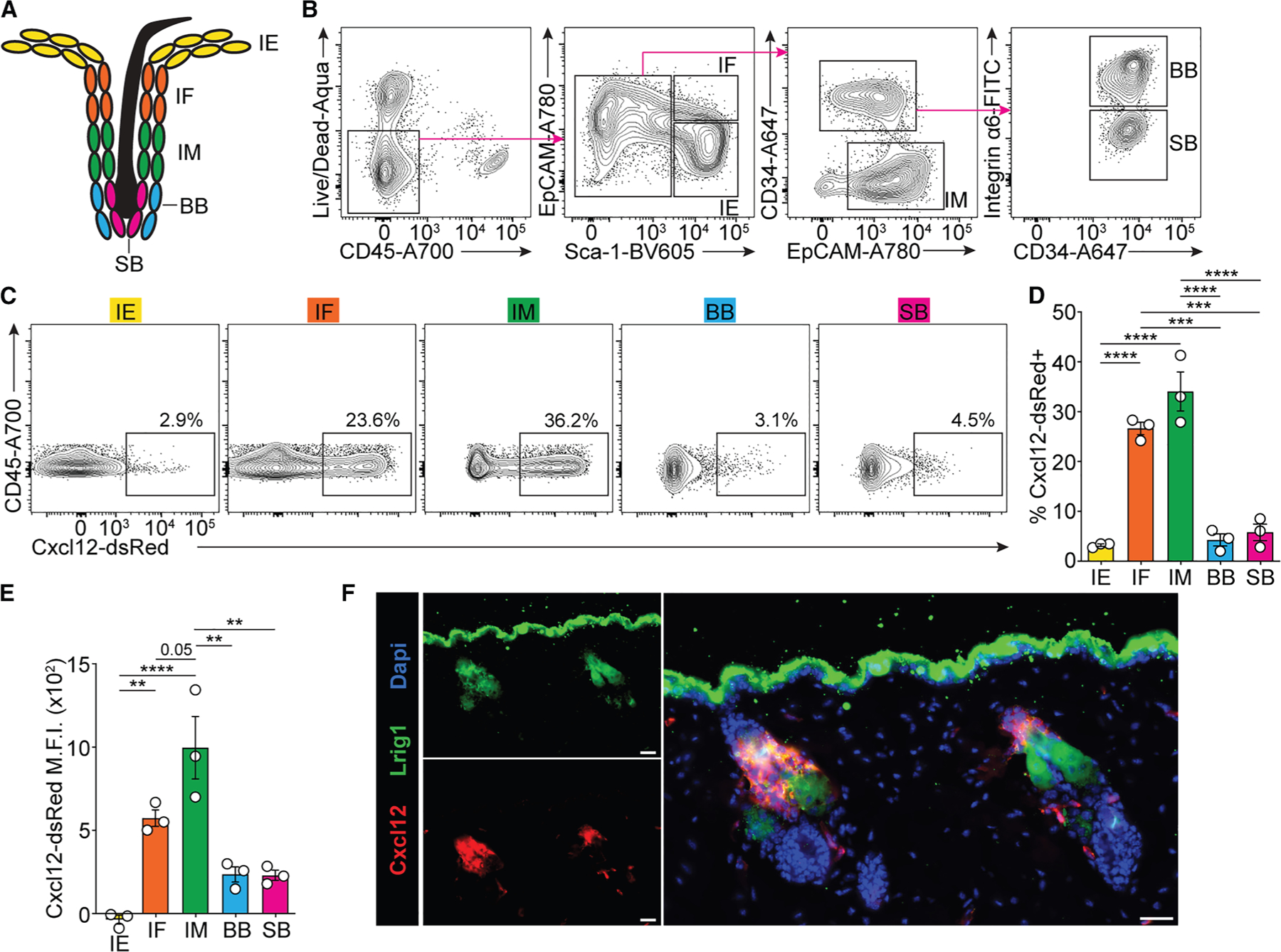
Cxcl12 is enriched in isthmic and infundibular keratinocytes of HFs (A) Rendering of skin epithelial subsets. IE, interfollicular epidermis; IF, infundibulum; IM, isthmus; BB, basal bulge; SB, suprabasal bulge. (B) Flow cytometry gating strategy for cutaneous epithelial subsets. (C) Representative flow cytometry plots of the percentage of Cxcl12-dsRed^+^ cells in each of the epithelial subsets. (D) Quantification of the percentage of Cxcl12-dsRed^+^ cells in cutaneous epithelial subsets. (E) Quantification of Cxcl12-dsRed expression level (M.F.I., mean fluorescence intensity) in cutaneous epithelial subsets. (F) Representative medium-power immunofluorescence images of skin tissue sections from Cxcl12-dsRed mice (scale bar: 50 μm). Data are representative of two independent experiments with similar results. Results are shown as individual data points and mean ± SEM (D and E). Statistics were calculated by one-way ANOVA with Tukey’s test for multiple comparisons. **p < 0.01, ***p < 0.001, ****p < 0.0001.

The HF is a complex cutaneous appendage that can be divided into distinct regions based on specific microanatomic landmarks (Figure 1A) and patterns of gene expression.^15,16^ Keratinocytes isolated from murine skin in the resting phase (telogen) of the HF cycle can be divided into at least five distinct subsets based on expression of certain cell surface markers (e.g., EpCAM, Sca-1, CD34, and CD49f [integrin α6]).^17^ These correspond to keratinocytes of the interfollicular epidermis (IE; Sca-1^+^ EpCAM^−^), HF infundibulum (Sca-1^+^ EpCAM^+^), HF isthmus (Sca-1^−^ EpCAM^+^ CD34^−^), HF basal bulge (Sca-1^−^ EpCAM^int^ CD34^+^ CD49f^+^), and HF suprabasal bulge (Sca-1^−^ EpCAM^int^ CD34^+^ CD49f^−^) (Figure 1B). Because a subset of skin Tregs localize around bulge HFSCs in adult mouse skin, where they promote HF growth,^2^ it was of significant interest to define the molecular pathways by which they traffic to this specialized niche. To initially address this question, we analyzed our previously generated bulk RNA sequencing data of sort-purified bulge HFSCs (Sca-1^−^ EpCAM^int^ CD34^+^) from murine skin^2^ and found that *Cxcl12* was the chemokine most highly expressed by these cells (Figure S1). To confirm expression of *Cxcl12* in bulge HFSCs, we isolated skin epithelium from Cxcl12-dsRed reporter mice and performed flow cytometry using the aforementioned markers to identify the different skin keratinocyte subsets. Surprisingly, we found that Cxcl12 was most highly enriched in keratinocytes of the HF infundibulum and isthmus, with lesser expression in the bulge HF keratinocytes where HFSCs reside (Figures 1C–1E). Cxcl12 was also expressed by subsets of blood endothelial cells and fibroblasts in the skin stroma, and minimal expression was observed in the cutaneous lymphoid and myeloid compartments (Figure S2). To directly visualize expression of Cxcl12 in the skin epithelium, immunofluorescence microscopy was performed on cryosections of telogen skin. This showed that Cxcl12 is expressed by keratinocytes in the infundibular and isthmic regions and that this expression overlaps with leucine-rich repeats and immunoglobulin like domain 1 (Lrig1), a marker that defines a subset of keratinocytes in the upper portion of the HF (Figure 1F).^18^ Collectively, these results demonstrate that keratinocytes in the upper HF are among the highest expressors of Cxcl12 in steady-state skin.

### Cxcr4 is preferentially expressed by skin Tregs

**Figure 2.**
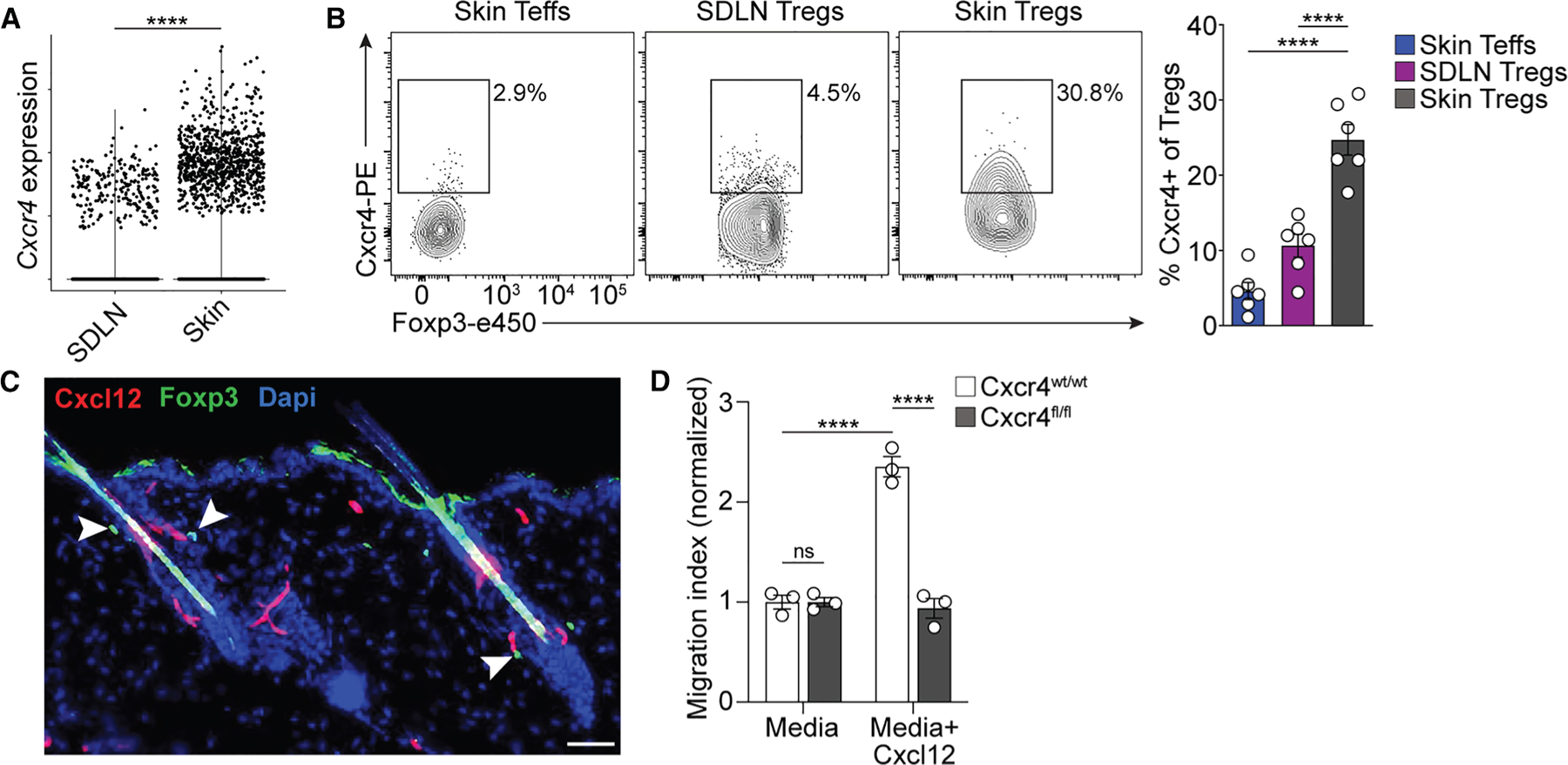
Cxcr4 is preferentially expressed by skin Tregs (A) Violin plot of *Cxcr4* mRNA expression in Tregs purified from the skin draining lymph node (SDLN) and skin. (B) Representative flow cytometry plots and quantification of the percentage of Cxcr4^+^ cells in each of the indicated CD4^+^ T cell subsets. (C) Representative medium-power immunofluorescence images of skin tissue sections from Cxcl12-dsRed × Foxp3-GFP mice (scale bar: 50 μm). (D) Quantification of Tregs from the indicated mice accumulating in the bottom well in a transmigration assay. Data in (A) are representative of one experiment. Data in (B)–(D) are representative of two independent experiments. Results are shown as individual data points (A) and individual data points and mean ± SEM (B and D). Statistics were calculated by unpaired Wilcoxon rank-sum test (A) or one-way ANOVA with Tukey’s test for multiple comparisons (B and D). ****p < 0.0001.

Cxcr4 is the only known cognate receptor for Cxcl12.^19^ We hypothesized that a subset of skin Tregs express Cxcr4 and use this receptor to accumulate around HFs. Therefore, we analyzed single-cell transcriptomic data of Tregs purified from skin and skin-draining LNs (SDLN)^20^ and found that *Cxcr4* mRNA was enriched in skin Tregs (Figure 2A). Flow cytometry analysis confirmed this finding at the protein level and showed that skin Tregs expressed significantly more Cxcr4 than effector CD4^+^ T cells (Teffs; CD4^+^Foxp3^−^) (Figure 2B). Additionally, immunofluorescence microscopy of telogen skin demonstrated Tregs in close proximity to Cxcl12^+^ cells in HFs (Figure 2C). To evaluate the capacity of Cxcr4 expression to facilitate chemotaxis to Cxcl12, sort-purified Tregs from pooled LNs and spleens of Foxp3-Cre × Cxcr4^wt/wt^ or Cxcr4^fl/fl^ mice were activated *in vitro* (to induce Cxcr4 expression) and used in transwell migration assays. After 4 hours of culture, there was a significant increase in the number of Tregs that had migrated in response to Cxcl12 compared to medium alone, which was completely abolished in the absence of Cxcr4 expression (Figure 2D). These data demonstrate that Cxcr4 is enriched on skin Tregs and that this expression promotes Cxcl12-dependent migration *ex vivo*.

### Cxcr4 on skin Tregs promotes accumulation in HF epithelium

To directly address whether skin Tregs utilize Cxcr4 to accumulate to HF epithelium *in vivo*, we generated Foxp3-Cre^ERT2^ × Cxcr4^fl/fl^ mice (Foxp3^ΔiCxcr4^) in which Cxcr4 can be inducibly deleted in Tregs with tamoxifen administration. We previously demonstrated that topical application of Z-4-hydroxytamoxifen (4OHT), the active metabolite of tamoxifen, to shaved back skin results in skin-specific genetic recombination in Tregs harboring a Foxp3-Cre^ERT2^ allele.^20^ Therefore, to selectively delete Cxcr4 on Tregs in skin, Foxp3^ΔiCxcr4^ mice were shaved in a “mohawk” configuration and treated with 4OHT on one side of back skin and the vehicle control (acetone only) on the contralateral side for 5 consecutive days (Figure S3A). Two days following the last topical treatment, skin was harvested, and cutaneous epithelium was enzymatically separated from the dermis. Flow cytometry analysis demonstrated that loss of Cxcr4 expression on Tregs isolated from epithelium of 4OHT-treated skin was associated with a trending decrease in the number of epithelium-associated Tregs (Figures S3B and S3C). To determine whether Cxcr4 expression on skin Tregs has functions other than localization, Foxp3^ΔiCxcr4^ mice were treated with tamoxifen for 2 weeks (Figure S3D). This treatment scheme ensured efficient Cxcr4 deletion on skin Tregs but did not perturb the number of these cells in skin (Figures S3E and S3F). Additionally, Cxcr4 deletion on Tregs did not alter the numbers of CD8^+^ T cells, Teffs, dendritic epidermal T cells, dermal γ/δ T cells, Ly6C^+^ monocytes, or neutrophils in skin (Figure S3G). These data suggest that Cxcr4 expression on skin Tregs may influence the localization to skin epithelium (including HFs) but does not impact homeostatic Treg accumulation in whole skin or influence cutaneous immune regulation.

**Figure 3.**
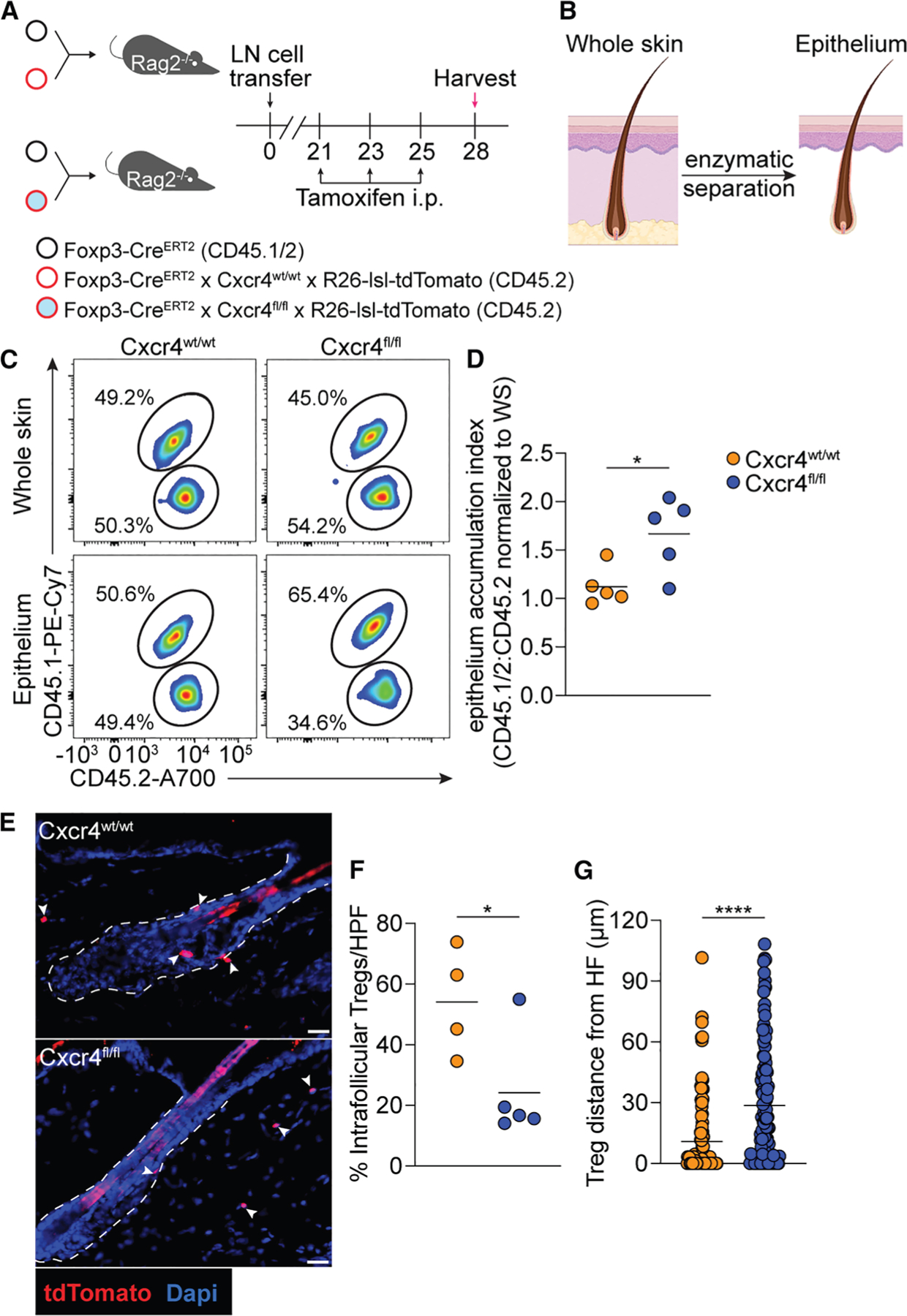
Cxcr4 promotes Treg accumulation to HFs (A) Schematic of the experiment in which lymph node (LN) cells from the indicated donor mice were mixed at a 1:1 ratio and transferred into *Rag2*^−/−^ recipient mice. After 3 weeks, recipient mice were treated with tamoxifen intraperitoneally (i.p.), and skin was harvested 3 days thereafter. (B) Rendering of enzymatic separation of skin epithelium, including HF epithelium, from the dermis. Created with BioRender. (C) Representative flow cytometry plots of the percentage of CD45.1/2^+^ and CD45.2^+^ Tregs (gated on live CD45^+^ TCRβ^+^ CD4^+^ CD8^−^ Foxp3− GFP^+^) from whole skin and skin epithelium following tamoxifen treatment. (D) Quantification of the ratio of CD45.1/2^+^: CD45.2^+^ epithelial Tregs normalized to the ratio observed in whole skin. (E) Representative medium-power immunofluorescence images of skin tissue sections from the indicated recipient mice. Dashed lines indicate boundary of HF epithelium (scale bar: 50 μm). (F) Quantification of the percentage of tdTomato^+^ Tregs in follicular epithelium. (G) Quantification of the distance of Tregs to the nearest HF epithelium. Data in (C)–(F) are representative of two independent experiments with similar results. Results are shown as individual data points and mean. Statistics were calculated by unpaired two-tailed Student’s t test. *p < 0.05, ****p < 0.0001.

Because the number of Tregs was not found to be significantly diminished in cutaneous epithelium following deletion of Cxcr4 on skin Tregs in the steady state, we posited that these cells may utilize other migratory receptors to access HF epithelium, since chemokine pathways are known to exhibit functional redundancy. To minimize potential compensatory mechanisms, we pursued an adoptive transfer approach, utilized extensively in the chemokine/chemokine receptor field, to address this issue.^21^ To do so, we generated Foxp3-Cre^ERT2^ × Cxcr4^wt/wt^ × Rosa26-lox-stop-lox (*LSL*)-tdTomato (Foxp3^iTom^) and Foxp3-Cre^ERT2^ x Cxcr4^fl/fl^ Rosa26-*LSL-*tdTomato (Foxp3^ΔiCxcr4–iTom^) mice. Total LN cells were isolated from these mice and mixed separately at an equal ratio with those from Foxp3-Cre^ERT2^ (Foxp3^ERT2^) mice (CD45.1/2) and transferred into *Rag2*^−/−^ mice, which are devoid of B and T cells (Figure 3A). After allowing sufficient time for lymphocytes to populate secondary lymphoid organs and fill peripheral tissue niches (i.e., 3 weeks^22^), mice were treated intraperitoneally with tamoxifen over the course of a week, which served to induce tdTomato expression in Foxp3^iTom^ and Foxp3^ΔiCxcr4–iTom^ mice and delete Cxcr4 in Tregs of the latter group. Subsequently, skin was harvested, and one portion was minced and enzymatically digested to liberate immune cells, while another portion underwent enzymatic separation of the cutaneous epithelium from the underlying dermis (Figure 3B). Flow cytometry analysis showed that a near-equal ratio of Tregs was maintained in whole skin and skin epithelium in the Foxp3^iTom^:Foxp3^ERT2^ recipient mice, whereas there was a significant decrease in the representation of Cxcr4^fl/fl^ Tregs in skin epithelium in the Foxp3^ΔiCxcr4–iTom^:Foxp3^ERT2^ group (Figures 3C and 3D). Immunofluorescence microscopy of tdTomato^+^ Tregs showed a decreased frequency of Tregs lacking Cxcr4 within follicular epithelium compared to those that were Cxcr4 sufficient (Figures 3E and 3F). Moreover, Cxcr4-sufficient Tregs were found in closer proximity to HF epithelium compared to those that were Cxcr4 deficient (Figure 3G). Collectively, these results suggest that skin Tregs utilize Cxcr4 to accumulate in and around HF epithelium.

### Glucocorticoid receptor signaling maintains Cxcr4 expression on skin Tregs

Given a putative role for Cxcr4 in guiding skin Tregs to HF epithelium, we next sought to determine the upstream signals that maintain expression of this chemokine receptor. Transforming growth factor β receptor (Tgfbr) signaling has been shown to induce Cxcr4 expression in CD4^+^ T cells.^23^ Additionally, Tgfbr signaling promotes Foxp3 expression and Treg development in specific experimental settings.^24,25^ To explore a potential contribution of Tgfbr signaling on Cxcr4 expression, the back skin of Foxp3-Cre^ERT2^ × Tgfbr2^fl/fl^ mice was shaved in a “mohawk” configuration and treated with topical 4OHT on one side and vehicle control on the contralateral side for 4 weeks (Figure S4A). Subsequent flow cytometry analysis showed no differences in skin Treg frequency or number when Tgfbr2 was deleted on these cells (Figures S4B and S4C), and no significant change in Foxp3 expression was observed (Figure S4D). Deletion of Tgfbr2 on skin Tregs was not associated with changes in the frequency of skin Teffs or CD8^+^ T cells (Figure S4E). Importantly, Tgfbr2-deficient skin Tregs did not show a diminished frequency or level of Cxcr4 expression compared to those in control-treated skin (Figures S4F and S4G). Therefore, Tgfbr signaling does not appear to sustain Cxcr4 expression on skin Tregs, is not required for maintenance of skin Treg identity, and appears to be dispensable for cutaneous immune homeostasis in the steady state.

**Figure 4.**
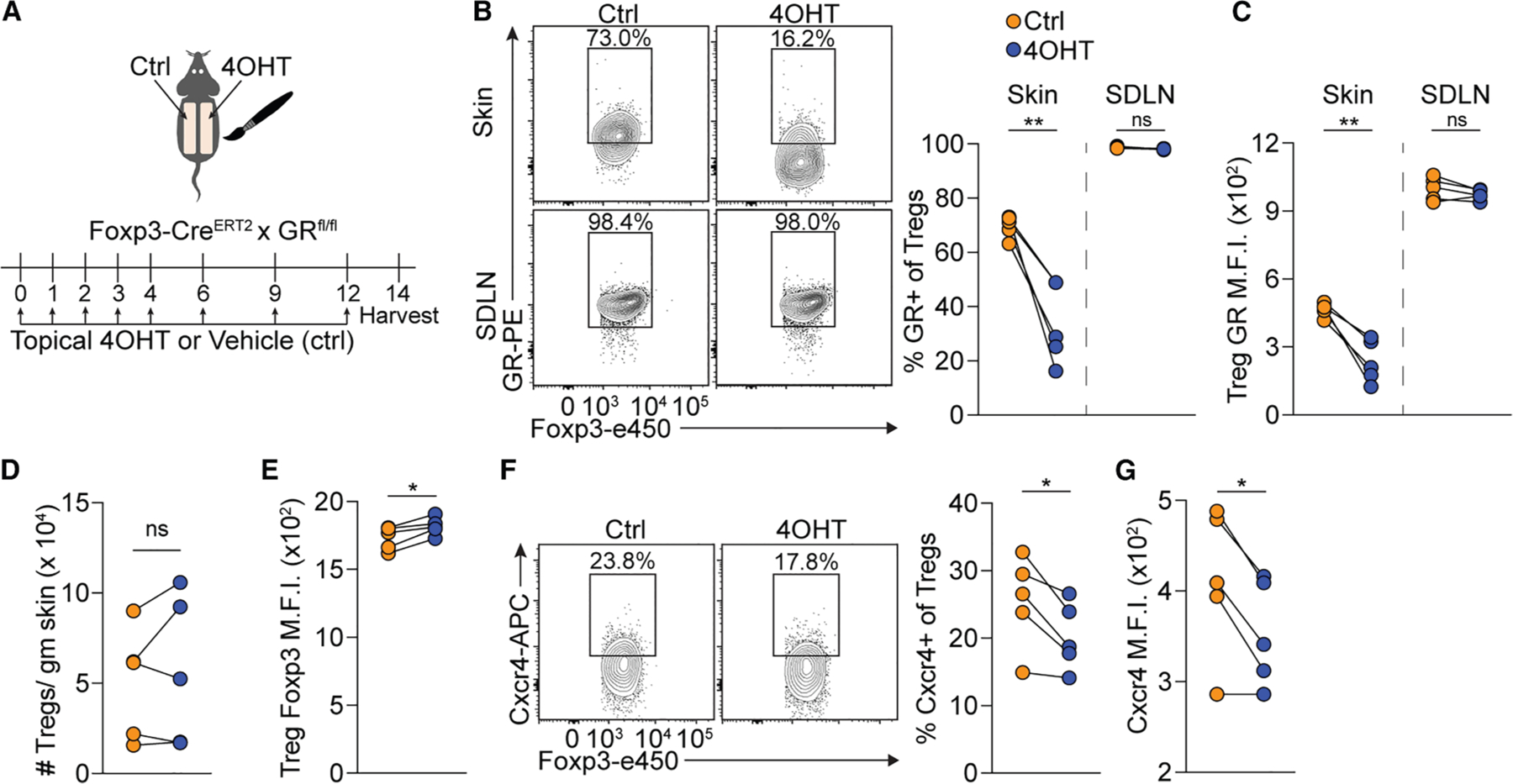
GR signaling in skin Tregs promotes Cxcr4 expression (A) Schematic of the experiment in which Foxp3^ΔiGR^ mice were treated for 5 consecutive days with 4OHT on one side of shaved back skin and vehicle control (acetone) on the contralateral side and every 3 days thereafter for 2 weeks, and skin and SDLNs were harvested thereafter. (B) Representative flow cytometry plots and quantification of the percentage of GR^+^ Tregs. (C) Quantification of M.F.I. of GR expression on skin and SDLN Tregs. (D) Quantification of the number of Tregs in skin. (E) Quantification of M.F.I. of Foxp3 expression on skin Tregs. (F) Representative flow cytometry plots and quantification of the percentage of Cxcr4^+^ Tregs. (G) Quantification of M.F.I. of Cxcr4 expression on skin Tregs. Data are representative of two independent experiments with similar results. Results are shown as paired data points. Statistics were calculated by paired two-tailed Student’s t test. *p < 0.05, **p < 0.01; ns, not significant.

Recently, GR signaling on Tregs was shown to stimulate HF growth in part by promoting expression of Tgfb3. Intriguingly, bulk RNA sequencing of GR-deficient and -sufficient skin Tregs showed Cxcr4 to be among the most differentially expressed genes.^5^ Therefore, we generated Foxp3-Cre^ERT2^ × GR^fl/fl^ mice, which were treated topically with 4OHT (or vehicle control) over 2 weeks, followed by harvest of skin and SDLNs (Figure 4A). Significant deletion of GR on skin Tregs was seen in 4OHT-treated compared to control-treated skin (Figures 4B and 4C). Importantly, topical 4OHT treatment did not impact GR expression on SDLN Tregs. Skin-specific GR loss in Tregs did not impact the number of skin Tregs (Figure 4D), in agreement with previous studies.^5^ Additionally, GR deletion in skin or SDLN Tregs did not diminish expression of Foxp3 and even slightly enhanced its expression (Figure 4E). However, skin-specific GR deletion on Tregs was associated with a decreased frequency of Cxcr4^+^ Tregs and diminished expression of Cxcr4 on these cells (Figures 4F and 4G). Overall, these data indicate that GR signaling in skin Tregs plays a role in maintaining Cxcr4 expression.

### Cxcr4 on skin Tregs promotes HF regrowth

**Figure 5.**
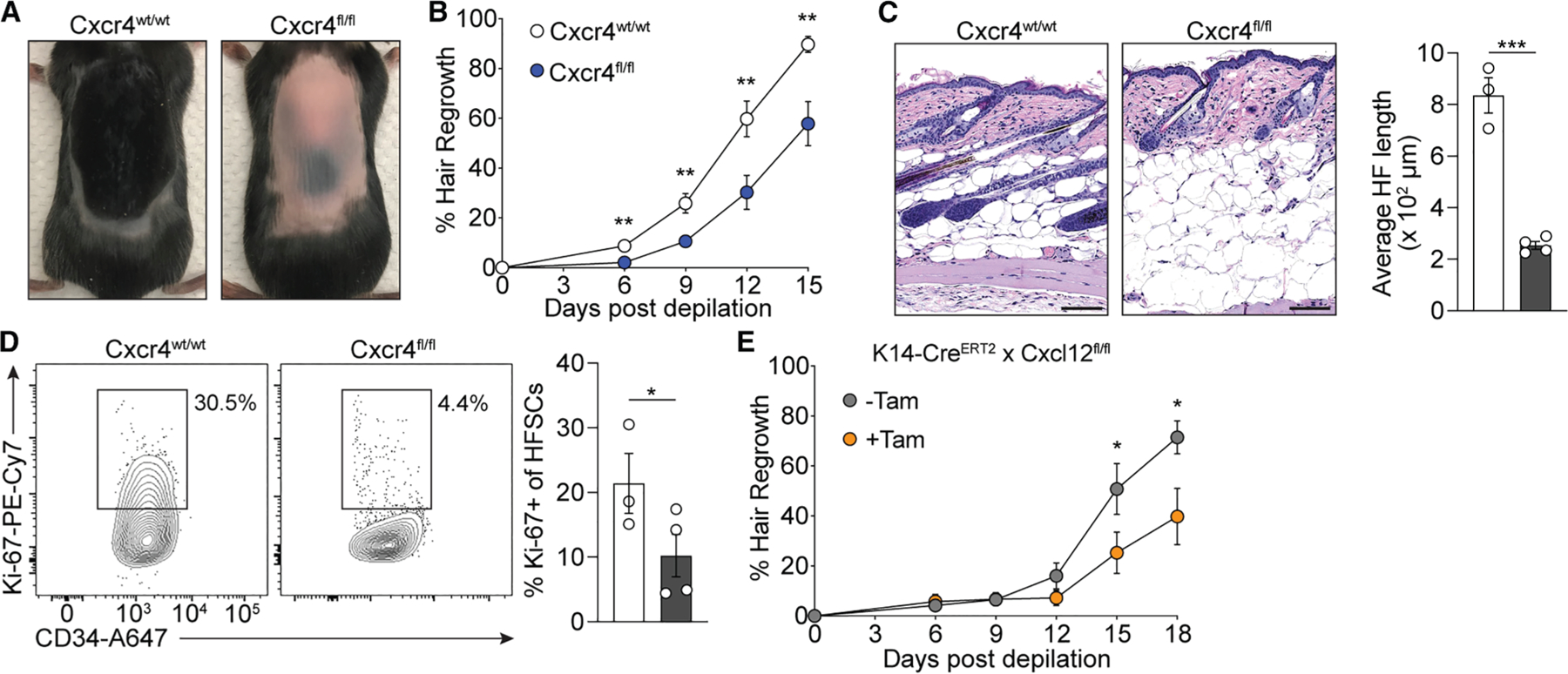
Cxcr4 on skin Tregs and Cxcl12 produced by keratinocytes coordinately mediate HF growth (A) Representative gross photographs of mouse back skin 12 days after chemical depilation. (B) Quantification of hair regrowth at defined time points following chemical depilation in Foxp3-Cre or Foxp3-Cre × Cxcr4-fl/fl mice. (C) Representative photomicrographs of skin tissue sections (left) and quantification of average HF length (right) from the indicated mice (scale bar: 100 μm). (D) Representative flow cytometry plots and quantification of the percentage of Ki-67^+^ HF stem cells (HFSCs; gated on live CD45^—^ Sca-1-EpCAM^int^ cells (Figure 1B)). (E) Quantification of hair regrowth at defined time points following chemical depilation in K14-Cre^ERT2^ × Cxcl12^fl/fl^ mice with or without tamoxifen treatment. Data are representative of two independent experiments with similar results. Results are shown as mean ± SEM (B and E) and individual data points and mean ± SEM (C and D). Statistics were calculated by unpaired two-tailed Student’s t test. *p < 0.05, **p < 0.01, ***p < 0.001.

Our results implicate the Cxcr4-Cxcl12 axis as a mechanism that promotes Treg localization to HF epithelium (Figure 3). We previously demonstrated that skin Tregs promote HF growth by stimulating HFSCs to proliferate and differentiate.^2^ Thus, we hypothesized that Cxcr4 expression on skin Tregs facilitates HF growth. To address this question, we depilated the dorsal back skin of adult Foxp3-Cre × Cxcr4^wt/wt^ and Foxp3-Cre × Cxcr4^fl/fl^ mice to synchronously induce the growth phase (anagen) of the HF cycle and tracked hair regrowth over time. This showed that mice lacking Cxcr4 expression on Tregs had a significant defect in hair regrowth (Figures 5A and 5B). Microscopically, HF length was significantly shorter in skin of mice lacking Cxcr4 on Tregs (Figure 5C), which was associated with decreased proliferation of HFSCs, as measured by the frequency of Ki-67-expressing cells (Figure 5D). Cytokeratin 14 (K14) is expressed by keratinocytes of the IE and in the upper portion of the HF, which includes the infundibulum and isthmus.^26^ Since keratinocytes in these regions express Cxcl12 (Figure 1), we reasoned that deletion of this chemokine may lead to diminished hair growth. Thus, K14-Cre^ERT2^ × Cxcl12^fl/fl^ mice (K14^ΔiCxcl12^) were treated with tamoxifen intraperitoneally (i.p.), and the back skin was depilated. Indeed, tamoxifen-treated K14^ΔiCxcl12^ mice showed delayed hair regrowth kinetics compared to non-tamoxifen-treated controls (Figure 5E). Hair regrowth was not influenced by the effects of tamoxifen, since mice treated with tamoxifen did not exhibit differences in hair regrowth compared to non-tamoxifen-treated controls (Figure S5). Overall, these data demonstrate that Treg expression of Cxcr4 and HF keratinocyte expression of Cxcl12 coordinately promote hair growth.

### Skin Tregs in healthy human scalp skin express CXCR4, and HF keratinocytes express cognate ligands

**Figure 6.**
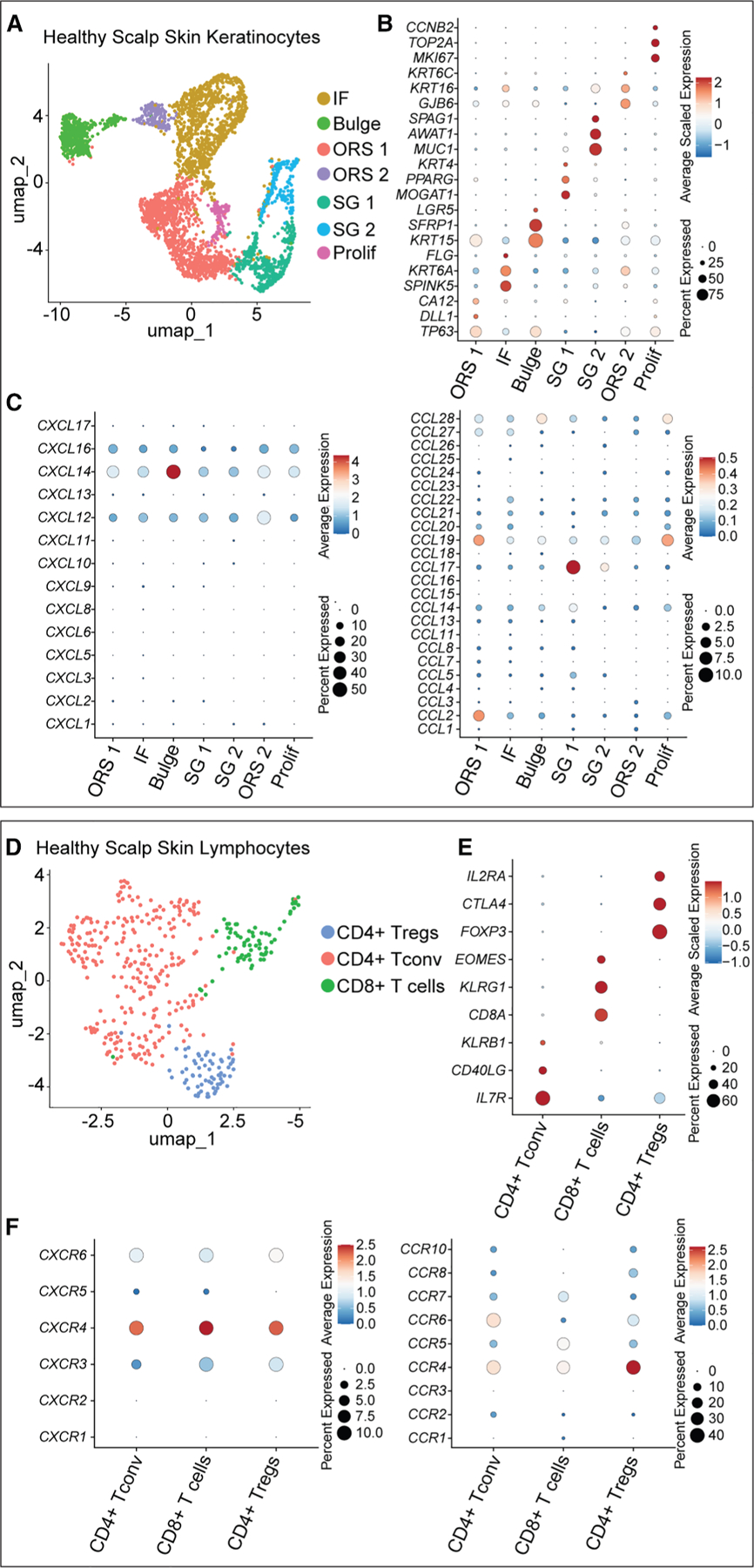
Human scalp skin demonstrates an enrichment of cognate CXCR4 chemokines in keratinocytes of the upper HF (A and D) Dimensional plots showing unsupervised clustering of keratinocytes (A) and lymphocytes (D) from fixed single-nucleus formalin-fixed paraffin-embedded sequencing of two samples of healthy scalp skin. (B and E) Dot plots of representative differentially expressed genes of distinct clusters of keratinocytes (B) and lymphocytes (E). (C and F) Dot plots of chemokine genes expressed by keratinocytes (C) and chemokine receptors expressed by lymphocytes (F) from healthy human scalp skin. Data are representative of two independent experiments.

The Cxcr4-Cxcl12 chemokine axis is an evolutionarily conserved mechanism of murine and human immune and stem cell accumulation in the bone marrow.^27,28^ We reasoned that this chemotactic pathway may have a similar pattern of expression in human skin compared to mouse skin (Figures 1 and 2). Therefore, we performed fixed single-nucleus RNA sequencing on two archived healthy human scalp skin specimens (Figure S6A; Table S1). Briefly, single nuclei were liberated from formalin-fixed paraffin-embedded tissue sections via enzymatic digestion and mechanical dissociation and underwent high-throughput RNA sequencing (Figure S6B), which resolved many of the cellular constituents found in skin (Figures S6C and S6D). Subclustering of keratinocytes revealed two clusters each of sebaceous gland and outer root sheath (ORS) keratinocytes and one cluster each of infundibular, bulge, and proliferating keratinocytes (Figures 6A and 6B). Analysis of chemokine transcripts revealed *CXCL12* to be most highly expressed by HF keratinocytes of the ORS with slightly lesser expression by other HF keratinocyte subsets (Figure 6C). Interestingly, *CXCL14*, another putative CXCR4 chemokine receptor ligand,^29,30^ showed enrichment in bulge HF keratinocytes. Among the remaining chemokines, *CCL17* was notable for its relatively high expression by sebaceous gland keratinocytes, whereas CCL2 and CCL19 showed moderate expression by ORS keratinocytes. Subclustering of lymphocytes resolved three distinct clusters, which included CD8^+^ T cells, CD4^+^ Teffs, and CD4^+^ Tregs (Figures 6D and 6E). Congruent with results from prior studies, Tregs in human scalp skin are enriched for *CCR4* and *CCR8* expression (Figure 6F).^31,32^ Additionally, we found that human skin Tregs, and other T cell subsets, also express relatively high levels of *CXCR4*.

To complement our findings, we analyzed a published single-cell RNA sequencing dataset containing 10 healthy human skin samples^33^ and subclustered the keratinocyte and T cell subsets (Figures S7A–S7D and S7F; Table S1). As was observed in our data, the CXCR4 ligands, *CXCL12* and *CXCL14*, were most highly expressed by a subset of ORS keratinocytes (Figure S7E). Interestingly, bulge keratinocytes also showed an enrichment of *CXCL12* expression in this reanalyzed dataset. In agreement with our findings, the reanalyzed dataset showed that CXCR4 is highly expressed across the major T cell subsets, including Tregs (Figure S7G). Collectively, these data suggest that human skin Tregs may utilize the *CXCR4*-*CXCL12* chemotactic pathway to accumulate to the upper HF.

## DISCUSSION

We show that Tregs in murine skin partially rely on Cxcr4 to accumulate around HF epithelium, which is mediated by Cxcl12 production by HF keratinocytes in the infundibular and isthmic regions. Furthermore, expression of *CXCL12* and *CXCL14* is enriched in keratinocytes of the upper HF in healthy human scalp skin, suggesting that this chemokine axis may be evolutionarily conserved. This pathway appears to be important to the accumulation of various immune cells and stem cells in specialized stromal niches. Stromal cells expressing Cxcl12 in murine and human bone marrow are necessary for maintenance of Cxcr4-expressing hematopoietic stem cells and long-lived plasma cells.^28,34^ Cxcr4 is also required for optimal accumulation of murine Tregs in the bone marrow, where they suppress autoantibody production by self-reactive B-1 cells.^35,36^ In murine skin, Cxcl12-expressing fibroblasts in the reticular dermis have been shown to recruit neutrophils and may play a role in interleukin-17 (IL-17)-mediated cutaneous dermatoses.^37^ Furthermore, CXCL12 is upregulated in certain human tumors and tumor-associated stromal cells, suggesting that induction of this chemokine could promote an immunosuppressive microenvironment by recruiting Tregs.^38^ Further investigation will be needed to reveal the impact of this pleotropic chemokine axis in health and disease.

In mice and humans, subsets of CD8^+^ and CD4^+^ T cells, including Tregs, localize to the upper HF and perifollicular region in skin.^2,3,39–41^ In adult mice, Treg localization to HFs is partially reliant on Cxcr4 expression, whereas, in the neonatal period, Ccl20 produced by HF keratinocytes in response to commensal microbial colonization of skin results in recruitment of CCR6^+^ Tregs.^42^ While speculative, the differences in chemokine receptor usage by Tregs to access HFs may be the result of distinct derivation of these cells. Whereas skin Tregs in the neonatal period appear to be derived from recent thymic emigrants,^42^ those in adult skin may originate from peripherally induced or activated Tregs in secondary lymphoid organs. Since a defect in skin epithelial Treg accumulation was uncovered in a competitive environment (Figure 3) and not in steady-state skin, we speculate that Tregs use additional chemotactic mechanisms to home to HFs, as functional redundancy is a known attribute of various chemokines and receptors. Furthermore, hair regrowth is slightly delayed when Cxcl12 is inducibly deleted from K14-expressing keratinocytes compared to constitutive deletion on Tregs (Figures 5B and 5E). One potential explanation for this observation is that Cxcl12 is also expressed by cutaneous blood endothelial cells (Figure S2), which likely serve as an additional source of Cxcr4 ligand that attracts a subset of Tregs to HFs (Figure 2C). Our single-nucleus RNA sequencing analysis of healthy human scalp skin suggests that skin Tregs may also utilize CCR4, CXCR6, and CCR10 to migrate toward CCL17, CXCL16, and CCL27 produced by HF keratinocytes, respectively (Figure 6). Treg accumulation to HFs likely plays a role in maintenance of these cells, as it has been shown that CD4^+^ T cell tissue residency is partially dependent on IL-7 expressed by the HFs.^39^ Thus, expression of CXCR4 and/or other chemokine receptors may act as a mechanism that results in provision of survival signals to Tregs in skin.

The non-classical functions of Tregs in skin are increasingly appreciated. Skin Tregs promote HF growth by modulating HFSC proliferation and differentiation by expression of Jag1 and Tgfb3, the latter of which is partially reliant on GR signaling.^2,5^ Furthermore, skin Tregs promote cutaneous barrier repair by promoting HFSC differentiation and migration into the wound site.^4^ Recently, it has also been shown that Treg localization to HFs also protects HFSCs from autoimmune attack.^20^ CD4^+^ T cell accumulation to HFs and sebaceous glands may also play a role in lipid homeostasis through thymic stromal lymphopoietin-induced adipose loss via sebum hypersecretion.^43^ Thus, skin CD4^+^ T cells serve a panoply of functions that modulate tissue regeneration and immune regulation of HF biology.

We find that murine isthmic and infundibular HF keratinocytes are enriched for Cxcl12 compared to other cutaneous keratinocyte subsets. This was a somewhat unexpected result, since bulk RNA sequencing of bulge HFSCs showed that *Cxcl12* was the highest expressed chemokine (Figure S1). The reason for this discrepancy is likely due to the relatively higher expression level of Cxcl12 by isthmic and infundibular HF keratinocytes compared to bulge HFSCs (Figure 1). Despite the lower Cxcl12 expression by bulge HFSCs, it may be that a subset of skin Tregs may localize to this specialize niche through this or other chemokine pathways. Indeed, direct Treg interactions with bulge HFSCs *in vitro* have been shown to induce proliferation of the latter cells.^44^ However, it is uncertain how accumulation of Tregs to the isthmic and infundibular region of HFs through the Cxcl12-Cxcr4 axis promotes HF growth, since this microanatomic niche is spatially distinct from the HF bulge. We speculate that skin Treg localization to the upper portion of the HF may induce hair growth through effects on intermediary cells that directly communicate with bulge HFSCs. Indeed, dermal fibroblasts have been shown to express bone morphogenic protein 2 (BMP2) and BMP4, which promote the refractory phase of telogen.^45^ Additionally, subsets of skin-resident macrophages can promote HF cycling through apoptotic cell death or maintain HF quiescence through expression of oncostatin M.^46,47^ Thus, it is conceivable that Tregs localizing to the upper HF indirectly modulate bulge HFSC activation through interactions with dermal fibroblasts and/or macrophages.

GR-mediated production of Tgfb3 by Tregs has been shown to promote HF growth.^5^ Transcriptomic analysis of GR-deficient skin Tregs revealed Cxcr4 to be among the most downregulated genes. However, due to the constitutive nature of GR deletion in this model, it was uncertain whether the hair regrowth defect was due to deleterious effects of Cxcr4 deletion during development and/or due to Cxcr4 deletion on Tregs systemically. We confirm that Cxcr4 expression on skin Tregs is significantly diminished at the protein level following inducible, skin-specific ablation of GR and provide additional evidence showing that GR signaling on skin Tregs is not required for the accumulation of these cells nor their role in maintaining cutaneous immune homeostasis. However, we leave open the possibility that, while GR deletion on skin Tregs is relatively efficient (Figures 4B and 4C), its expression is not completely abolished and that the small subset of remaining GR-expressing Tregs may have the capacity to preserve immune homeostasis. Glucocorticoids are produced by adrenal glands during the steady state in a diurnal fashion, where they have been shown to influence T cell distribution in tissues through upregulation of Cxcr4.^48^ Glucocorticoids increase in skin following chemical depilation but not in the peripheral blood, suggesting that local, *de novo* production of gluococorticoids is critical for optimal hair regrowth.^5^ Additionally, emerging evidence shows that keratinocytes in skin produce endogenous glucocorticoids through expression of enzymes, such as Cyp11b1, which has been shown to modulate immune responses.^49^ It will be fascinating for future studies to functionally dissect the importance of keratinocyte-derived endogenous or exogenously provided glucocorticoids on skin Treg biology in the context of hair regeneration.

Collectively, we demonstrate that skin Tregs promote hair growth via GR-mediated Cxcr4 expression. We find that the Cxcr4-Cxcl12 chemokine axis regulates immune-stem cell crosstalk in a barrier tissue, which fine-tunes a regenerative response. Additionally, we show that similar chemokine-chemokine receptor combinations are expressed by skin Tregs and HF keratinocytes in human skin. However, since the phenotype of inducible Cxcr4 ablation on Tregs is relatively modest, strategies aimed at augmenting Treg trafficking in skin may need to selectively target combinations of chemokine receptors. Given the difficulty of treating different forms of alopecia, and since Tregs appear to play an important role in promoting tissue regeneration, it will be of significant interest to develop therapeutics that can attract Tregs to HFs.

### Limitations of the study

Although we show that Cxcr4 expression on Tregs is important for HF regrowth, we used a constitutive Cre that deletes Cxcr4 in Tregs systemically and throughout development. Therefore, the effect on hair regrowth may be more subtle if Cxcr4 deletion were only to occur in skin Tregs in adult mice. We show that murine isthmic/infundibular HF keratinocytes are enriched for expression of Cxcl12 and show delayed hair regrowth when this chemokine is inducibly ablated in keratinocytes of the upper portion of the HF using the K14-CreERT2 strain. However, while we show that CD34^+^ HFSCs express less Cxcl12 mRNA, a potential contribution of this cell type on Treg accumulation to HF epithelium and stimulation of HF regrowth was unexplored. Finally, while we characterized chemokine receptor and ligand expression in human skin Tregs and keratinocytes using single fixed nuclear RNA sequencing, functional experiments that manipulate the CXCR4-CXCL12 pathway in *ex vivo* human skin and/or HFs were lacking.

## RESOURCE AVAILABILITY

### Lead contact

Requests for further information, resources, and reagents should be directed to and will be fulfilled by the lead contact, Michael D. Rosenblum (michael.rosenblum@ucsf.edu).

### Materials availability

Reagents and resources can be shared upon request.

## STAR★METHODS

### EXPERIMENTAL MODEL AND STUDY PARTICIPANT DETAILS

#### Healthy human scalp skin samples

The procurement of archived patient formalin-fixed paraffin-embedded tissue (FFPE) was approved by the Institutional Review Board at the University of California, San Francisco (UCSF) (21–33678) and was conducted according to the Declaration of Helsinki. A waiver of informed consent was granted as this was a retrospective analysis of left-over archival specimens and no study procedures were performed. Two healthy human scalp skin samples were retrieved from the UCSF Dermatopathology Service archives, and H&E slides were reviewed by an expert dermatopathologist (J.N.C.). The patient clinical characteristics are documented in Table S1. The influence of sex on the results is uncertain given the small sample size, which is a limitation we acknowledge.

#### Animals

All mice were bred and maintained in a specific pathogen free mouse facility in accordance with the Laboratory Animal Resource Center and Institutional Animal Care and Use Committee of the University of California San Francisco (UCSF). Mice were socially housed under a 12-h day/night cycle. 7–10 week old mice in the second telogen of the hair follicle cycle were used for all experiments. Group-housed littermate controls were used for all experiments, and animals of both sexes were included. Sex, parental cage, and weaning cage were randomized among experimental groups. Strains used in this study include Foxp3-Cre^YFP^ (Foxp3^tm4(YFP/icre)Ayr^); Foxp3-Cre^ERT2–GFP^ (Foxp3tm9(EGFP/cre/ERT2)Ayr); Foxp3-GFP (Foxp3^tm2Tch^); Rosa26tdTomato (Gt(ROSA)26Sortm14(CAG-tdTomato)Hze); K14-Cre^ERT2^ (KRT14^(Cre/ERT2)1lpc/MtzJ^); GR^fl/fl^ (Nr3c1^tm1.1Jda^); Cxcr4^fl/fl^ (Cxcr4^tm2Yzo^), Cxcl12^fl/fl^ (Cxcl12^tm1.1Link^); Tgfbr2^fl/fl^ (Tgfbr2^tm1Karl^); Cxcl12-dsRed (Cxcl12^tm2.1Sjm^); CD45.1 (Ptprc^a^ Pepc^b^); Rag2^−/−^ (Rag2^tm1.1Cgn^).

### METHOD DETAILS

#### Administration of tamoxifen and 4OHT

Tamoxifen (Sigma-Aldrich) was dissolved in corn oil (Sigma-Aldrich) at 37°C by shaking overnight. For Cre recombinase induction in Cre^ERT2^-expressing mouse strains, Tamoxifen was administered intraperitoneally at a dose of 100 mg/kg. In experiments using topical 4OHT (Sigma-Aldrich), 5 mg of 4OHT was dissolved in 10 mL of acetone (Sigma-Aldrich) to generate a stock concentration of 500 μg/mL. 4OHT was further diluted with acetone to generate a working concentration of 50 μg/mL (1:10 dilution). 100 μL of 4OHT/acetone (50 μg/mL) was then applied to anesthetized mice in a dropwise manner to one side of exposed dorsal back skin of mice shaved in a mohawk configuration to cover the entire shaved skin surface (no depilation is performed). Acetone alone was applied in the same manner to the contralateral side of shaved dorsal back skin. This procedure is repeated for 5 consecutive days and then applied every two to three days for 2–4 weeks.

#### Anagen induction

Dorsal back hair was shaved with clippers before applying depilatory cream (Nair) to the shaved region for 30 s before wiping clean. For monitoring of clinical hair regrowth, standardized pictures were taken on the day of depilation (day 0) and then at the indicated time points until day 15 or 18. Anagen induction was quantified using intensity analysis on ImageJ software (v1.46r, NIH, USA) at each time point or as a percent of pigmented dorsal skin relative to baseline (day 0).

#### Adoptive transfer of lymph node cells

Mice were euthanized, and inguinal, brachial, axillary, and mesenteric lymph nodes were harvested, pooled, and mashed through a 40 μm strainer using a 5 mL syringe plunger and strained into cold RPMI containing 10% calf serum, 1% HEPES, 1% non-essential amino acids, 1% GlutaMAX, and 1% penicillin-streptomycin. Cells were spun down, washed twice with PBS, and resuspended in PBS. Cells were counted and resuspended at 1 × 10^8^/mL in sterile PBS (Gibco) and 100 μL (1 × 10^7^ LN cells) were injected retroorbitally per recipient mouse.

#### Transwell migration assay

Skin-draining lymph nodes, mesenteric lymph nodes, and spleen cells from Foxp3-Cre^YFP^ or Foxp3-Cre^YFP^ x Cxcr4^fl/fl^ mice were pooled and mashed through a 40 μm strainer using a 5 mL syringe plunger and strained into cold RPMI containing 10% calf serum, 1% HEPES, 1% non-essential amino acids, 1% GlutaMAX, and 1% penicillin-streptomycin. Cells were spun down and resuspended in 500 μL red blood cell lysis buffer (Thermo Fisher) for 3 min. CD4^+^ T cells were isolated by negative selection using an EasySep Mouse CD4 T cell isolation kit (StemCell Technologies). Live CD45^+^ CD4^+^ YFP+ cells were then sorted on a BD FACS Aria2.

Sort-purified Foxp3-YFP+ cells were plated in 48-well flat bottom plates at 1 × 10^6^ cells in 1 mL of complete DMEM with IL-2 (2000 U/ml, Tonbo Biosciences) and stimulated with mouse anti-CD3/CD28 beads at cells:beads ratio of 1:3 (Dynabeads, Thermo Fisher Scientific). After five days of culture, 5 × 10^5^ cells in 100 μL of complete DMEM were added to the upper chamber of transwell plates and complete DMEM media only or media with recombinant murine Cxcl12 (100 ng/mL (R&D)) was added into the lower chamber. Plates were incubated at 37°C for 48 h and media in the lower chamber was collected. Cells were then stained for flow cytometry (live CD45^+^ CD4^+^ Foxp3+) and counted using Absolute Bright Counting beads (Thermo Fisher).

#### Tissue processing

After euthanasia, whole back skin was shaved and dissected. Any remaining scapular or inguinal fat was removed, and the remaining tissue was weighed, finely minced with scissors, placed in a 50 mL conical containing 3 mL of digestion media (2 mg/mL collagenase XI, 0.5 mg/mL hyaluronidase, 0.1 mg/mL DNase in RPMI with 10% calf serum, 1% HEPES, 1% non-essential amino acids, 1% GlutaMAX, and 1% penicillin-streptomycin), and digested in a bacterial shaker for 40 min at 37°C and 180 rpm. Digestion was then quenched with 8 mL of cold RPMI and samples were vortexed for 10 s and strained through 100 μm strainers. The resulting single cell suspension was then plated in 96-well plates for staining. For LN samples, axillary, brachial, and inguinal LNs were dissected and mashed through a 40 μm filter with a 5 mL syringe plunger into cold media. For cutaneous epithelial cell preparations for flow cytometric analysis, 2.25 cm^2^ of back skin was harvested and defatted mechanically using forceps. Skin was placed dermis side down in a well of a 12-well plate with 1 mL of Thermolysin (0.20 mg/mL; Sigma) and placed in a 37°C incubator for 1 h. Skin epithelium was gently disassociated from the underlying dermis in a Petri dish containing 1 mL RPMI/HEPES/P-S/FCS media using forceps and minced with scissors. Media containing epithelial fragments was pipetted up and down using a P1000 pipette 20 times before filtering through 100 μm strainers. Cells were counted and stained for flow cytometry.

#### Flow cytometry

Single-cell suspensions were pelleted and resuspended in PBS with 2% FBS containing fluorophore-conjugated antibodies listed in the key resources table. Cells were initially stained with antibodies targeting cell surface proteins and Ghost 510 viability dye (Tonbo Biosciences) for 30 min. For intracellular staining, cells were then fixed and permeabilized using the Cytofix/Cytoperm kit (BD Biosciences). Samples were run for analysis on a BD Fortessa or sorted on a BD FACS Aria2.

For all cell types, initial forward scatter vs. side-scatter gates were carefully adjusted by backgating on live CD45^+^ and/or live CD45^−^populations to include all cells and exclude debris. Strict dead cell and doublet exclusion was performed prior to gating for immune cells (CD45^+^) and/or epithelial cells (CD45^−^). αβT cells were gated as CD45^+^ TCRβ+ and subsetted into Teffs (CD4+ FoxP3-), Tregs (CD4+FoxP3+), CD8 T cells (CD8^+^). Non-αβT cells were gated as CD45^+^ CD3^+^ γδ+ and subsetted into dermal γδ T cells (TCRβ− CD3^mid^) and dendritic epidermal T cells (DETCs, TCRβ− CD3^hi^). Cxcl12-dsRed reporter mice were used to report Cxcl12 expression. Macrophages (CD64^+^ CD11b+ CD11c−) and dendritic cells (CD64-MHCII+CD11c+) were gated after excluding T cells, eosinophils (CD45^+^ Ly6G− CD11b+ Siglec F+), and neutrophils (CD45^+^ Ly6G + MHC II-). Skin epithelial cells were gated as live CD45^−^cells, and subsetted according to expression of Sca-1, EpCAM, and CD34. Cell numbers were calculated by using CountBright Absolute counting beads (Thermo Fisher).

#### Routine histology and immunofluorescence microscopy

For routine histology, murine dorsal back skin strips (∼4–5 mm width x 10–20 mm length) were fixed in 10% neutral-buffered formalin and paraffin-embedded. Tissue was stained with hematoxylin and eosin (H&E) by the University of California, San Francisco Dermatopathology Service. Slides were scanned at 40x resolution with an Aperio AT2 scanner (Leica Biosystems) using a 20x/0.75NA Plan Apo objective with a 2× optical mag changer to generate digital images. Image resolution was 40x: 0.25 μm/pixel. Slides were viewed digitally using QuPath software.^50^ Quantification of the hair follicle length was performed by an expert dermatopathologist (J.N.C.).

For immunofluorescent tissue staining, shaved back skin strips from Cxcl12-dsRed x Foxp3-GFP, and Foxp3^iTom^ and Foxp3^ΔiCxcr4–iTom^ mice were fixed in 2% paraformaldehyde on a rocker at room temperature for 4 h. After washing in PBS, tissues were embedded in optical cutting temperature compound (OCT) and flash-frozen in isopentane (Sigma) cooled with dry ice. 10 μm sections were cut on a cryostat (ThermoFisher Cryostar NX50) onto SuperFrost Plus slides. Tissue sections were air-dried for one hour, washed in PBS, and mounted in ProLong Gold antifade reagent with DAPI (ThermoFisher). Tissues were imaged on a Keyence Fluorescence BZ-X810 microscope. The number of tdTomato+ Tregs within hair follicle epithelium was quantified per 200x high power field (HFP) across 5 randomly HFPs per tissue section. The distance of tdTomato+ Tregs to the nearest hair follicle epithelium was also quantified across 5 randomly HPFs per tissue section.

#### Fixed single nuclear RNA sequencing from archival human scalp skin specimens (snFFPEseq)

FFPE tissue blocks for each sample were retrieved. Fixed nuclei were isolated according to a demonstrated 10x Genomics protocol (CG000632, Rev C). Briefly, four 40 μm tissue scrolls per sample were sectioned into GentleMacs C tubes (Miltenyi). Samples were deparaffinized using xylene, ethanol, and water immersion. 2 mL of enzyme dissociation mix (Liberase TH (1mg/mL, Millipore Sigma) in RPMI) was added a GentleMacs C tube containing the deparaffinized tissue. Tubes were loaded into a GentleMacs Octodissociator with heaters and tissue was dissociated using the 37C_FFPE_1 program. Following enzyme digestion and mechanical dissociation, tubes were spun at 300 rcf for 1 min. Subsequently, the pellets containing nuclei were resuspended in the supernatant and were pipetted through a sterile 30 μm strainer (Miltenyi), followed by a wash with 2 mL of chilled PBS into a 15 mL conical tube. Tubes were spun down at 850 rcf for 5 min and resuspended in 500 μL of tissue resuspension buffer (50 mM Tris buffer/0.24 U/μL RNAse inhibitor/PBS). Nuclei were counted using Celleca counter.

Samples were run on separate lanes of a 10X Chromium chip with GEM-X Flex v2 kit (10X Genomics) following manufacturer instructions by the UCSF Institute for Human Genetics Sequencing Core. Libraries were sequenced on an Illumina NovaSeq X. Fastq files were aligned to the GENCODE v44 human reference genome and barcode matrices were generated using Cellranger 7.0.

Downstream data analysis, including clustering, visualizations, and exploratory analyses, were performed using Seurat R package 5.1.0. Nuclei data from each sample were merged and underwent preprocessing by filtering on the following parameters: <700 or >7000 features, >3,000 reads, or >10% mitochondrial genes were filtered out. The merged dataset was normalized and scaled. PCA and UMAP were run on the merged object, and an initial low-resolution clustering was generated using the first 20 principal components. Based on this initial clustering, we chose to use the first 18 principal components and a resolution of 0.2, using the *Harmony* integration function. Markers for each cluster were identified with the Seurat *FindAllMarkers* function (Wilcoxon rank-sum test, min.pct = 0.25, only.pos = True, thresh.use = 0.25), and cells were annotated according to known expression of differentially expressed genes (DEGs). Log-normalized gene expression data were used for visualizations with UMAP plots (FeaturePlot). Keratinocyte and lymphocyte clusters were separately subclustered, normalized and scaled, and initial low-resolution clustering was generated using the first 25 principal components. We used the *Harmony* integration function and chose to use the first 16 principal components and a resolution of 0.2 for the keratinocyte subcluster and the first 21 principal components and a resolution of 0.3 for the lymphocyte subcluster.

### QUANTIFICATION AND STATISTICAL ANALYSIS

Statistical analyses were performed using Prism software package 9.0 (GraphPad). *p* values were calculated using two-tailed unpaired or paired Student’s t-test or one-way ANOVA and as indicated in the Figure Legends. Mice cohort size was designed to be sufficient to enable accurate determination of statistical significance and no animals were excluded from the statistical analysis. Mice were assigned to treatment or control groups randomly. Apart from scRNAseq and spatial transcriptomics, all experiments were repeated 2–3 times, and all data points represent individual biological replicates.

## Supplementary Material

1

SUPPLEMENTAL INFORMATION

Supplemental information can be found online at https://doi.org/10.1016/j.celrep.2025.116467.

## Figures and Tables

**Table:** KEY RESOURCES TABLE

REAGENT or RESOURCE	SOURCE	IDENTIFIER
Antibodies		

CD45 Alexa 700 Conjugated (30-F11) (1/200)	Thermo Fisher	Cat# 56–0451-82, RRID:AB_891454
EpCAM APC-eFluor 780 Conjugated (G8.8) (1/200)	Thermo Fisher	Cat# 47–5791-82, RRID:AB_2573986
Sca-1 BV605 Conjugated (D7) (1/200)	BioLegend	Cat# 108133, RRID:AB_2562275
CD34 Alexa 647 Conjugated (RAM34) (1/200)	BD Biosciences	Cat# 560230, RRID:AB_1645200
CD49f FITC Conjugated (eBio-GO83) (1/200)	Thermo Fisher	Cat# 11–0495-82, RRID:AB_11150059
FOXP3 e450 Conjugated (FJK-16) (1/100)	Thermo Fisher	Cat# 48–5773-82, RRID:AB_1518812
CD45 Conjugated (30-F11) (1/200)	Thermo Fisher	Cat# 56–0451-82, RRID:AB_891454
CD45.1 PE-Cy7 Conjugated (A20) (1/200)	BioLegend	Cat# 110729, RRID:AB_1134170
CD45.2 Alexa 700 Conjugated (104) (1/200)	Thermo Fisher	Cat# 56–0454-82, RRID:AB_657752
Glucocorticoid receptor (GR) PE Conjugated (G-5) (1/100)	Santa Cruz Biotechnology	Cat# sc-393232, RRID:AB_2687823
CXCR4 PE Conjugated (L276F12) (1/100)	BioLegend	Cat# 146506, RRID:AB_2562783
CXCR4 APC Conjugated (L276F12) (1/100)	BioLegend	Cat# 146507, RRID:AB_2562784
CD3E PerCP Conjugated (145–2C11) (1/100)	BD Biosciences	Cat# 561089, RRID:AB_10584323
TCR beta APC Conjugated (H57–597) (1/200)	Thermo Fisher	Cat# 17–5961-81, RRID:AB_469480
TCR gamma/delta APC Conjugated (GL3) (1/400)	Thermo Fisher	Cat# 17–5711-82, RRID:AB_842756
CD8 BV785 Conjugated (53–6.7) (1/200)	BioLegend	Cat# 100750, RRID:AB_2562610
CD4 BV650 Conjugated (RM4–5) (1/200)	BD Biosciences	Cat# 563747, RRID:AB_2716859
CD4 BV605 Conjugated (RM4–5) (1/200)	BioLegend	Cat# 100548, RRID:AB_2563054
Ki-67 PE-Cy7 Conjugated (B56) (1/100)	BD Biosciences	Cat# 561283, RRID:AB_10716060
MHC II e450 Conjugated (M5/114.15.2) (1/400)	Thermo Fisher	Cat# 48–5321-82, RRID:AB_1272204
Podoplanin PE-Cy7 Conjugated (8.1.1) (1/200)	Thermo Fisher	Cat# 25–5381-82, RRID:AB_2573460
Ly6G PE-Cy7 Conjugated (1A8) (1/400)	BioLegend	Cat# 127618, RRID:AB_1877261
CD64 BV786 Conjugated (X54–5/7.1) (1/100)	BD Biosciences	Cat# 741024, RRID:AB_2740644
CD11c PerCP-Cy5.5 Conjugated (N418) (1/200)	BioLegend	Cat# 117327, RRID:AB_2129642
CD11b APC-eFluor 780 Conjugated (M1/70) (1/200)	Thermo Fisher	Cat# 47–0112-82, RRID:AB_1603193
Lrig1 Unconjugated (polyclonal) (1/500)	R&D	Cat#AF3688
Alexa Fluor^®^ 647 AffiniPure^®^ Donkey Anti-Goat IgG (H + L)	Jackson Immunoresearch	Cat#705–605-003

Chemicals, peptides, and recombinant proteins		

recombinant murine Cxcl12	R&D	Cat#460-SD
recombinant human IL-2	Tonbo Biosciences	Cat#21–8029-U050
Tamoxifen	Sigma-Aldrich	Cat#T5648
(Z)-4-Hydroxytamoxifen	Sigma-Aldrich	Cat#H7904
Acetone	Sigma-Aldrich	Cat#179124
Collagenase	Sigma-Aldrich	Cat#C9407
Hyaluronidase	Sigma-Aldrich	Cat#C9407
DNAse	Sigma-Aldrich	Cat#DN25
Fetal calf serum	Fisher scientific	Cat#SH3007303
eBioscience^™^ 10X RBC Lysis Buffer	Thermo Fisher	Cat#00–4300-54
Thermolysin	Sigma-Aldrich	Cat#T7902
OCT compound	VWR International	Cat#25608–930
16% paraformaldehyde	VWR International	Cat#15710
2-methylbutane	Sigma-Aldrich	Cat#M32631
Liberase-TH	Sigma-Aldrich	Cat#5401135001
Men Body Cream Hair Remover	Nair	SKU#00022600003045

Critical commercial assays		

FoxP3/Transcription factor staining buffer	Thermo Fisher	Cat#11500597
Ghost Dye^™^ Violet 510 Live/Dead Stain	Tonbo Biosciences	Cat#13–0870-T100
EasySep^™^ Mouse CD4 T cell Negative Selection Kit	StemCell Technologies	Cat#19852
Dynabeads^™^ Mouse anti-CD3/CD28	Thermo Fisher	Cat#11456D

Deposited data		

Healthy human scalp skin fixed single nuclear RNA sequencing	This paper	GEO accession number: GSE305547
Healthy human scalp skin single cell RNA sequencing	Ober-Reynolds et al.^33^	github.com/GreenleafLab/scScalpChromatin
Mouse skin and skin-draining lymph node Treg single cell RNA sequencing	Cohen et al.^20^	GEO accession numbers: GSE175746 and GSE136160
Mouse hair follicle stem cell bulk RNA sequencing	Ali et al.^2^	GEO accession number: GSE76102

Experimental models: Organisms/strains		

*Foxp3*^*YFP*—*cre*^ mice	Jackson Laboratory	Mouse: Foxp3-Cre^YFP^: B6.129(Cg)-*Foxp3*^*tm4(YFP/icre)Ayr*^/J
*Foxp3^CreERT2-GFP^* mice	Jackson Laboratory	Mouse: Foxp3-Cre^ERT2^:
		*Foxp3tm9(EGFP/cre/ERT2)Ayr*/J
*Foxp3-GFP* mice	Jackson Laboratory	Mouse: Foxp3-GFP: C.Cg-*Foxp3*^*tm2Tch*^/J
*Rosa26-lox-stop-lox-tdTomato* mice	Jackson Laboratory	Mouse: Rosa26^iTom^: B6.Cg-*Gt(ROSA)26Sor^tm14(CAG-tdTomato)Hze^*/J
*Keratin14-Cre*^*ERT2*^ mice	Jackson Laboratory	Mouse: K14-Cre^ERT2^: B6.Cg-Tg(KRT14-cre/ERT2)1Ipc/MtzJ
*Nr3c1-flox* mice	Jackson Laboratory	Mouse: GR^fl/fl^: B6.129S6-*Nr3c1*^*tm2.1Ljm*^/J
*Cxcr4-flox* mice	Jackson Laboratory	Mouse: Cxcr4^fl/fl^: B6.129P2-*Cxcr4*^*tm2Yzo*^/J
*Cxcl12-flox* mice	Jackson Laboratory	Mouse: Cxcl12^fl/fl^: B6(FVB)-*Cxcl12*^*tm1.1Link*^/J
*Tgfbr2-flox* mice	Jackson Laboratory	Mouse: Tgfbr2^fl/fl^: B6;129-*Tgfbr2*^*tm1Karl*^/J
*Cxcl12-dsRed* mice	Jackson Laboratory	Mouse: Cxcl12-dsRed: *Cxcl12*^*tm2.1Sjm*^/J
*CD45.1* mice	Jackson Laboratory	Mouse: CD45.1: B6.SJL-*Ptprc*^*a*^ *Pepc*^*b*^/BoyJ
*Rag2-knockout* mice	Jackson Laboratory	Mouse: Rag2^−/−^: B6.Cg-*Rag2*^*tm1.1Cgn*^/J

Software and algorithms		

GraphPad Prism	GraphPad Software, Inc	http://www.graphpad.com/scientific-software/prism/
FlowJo	FlowJo, LLC	https://www.flowjo.com/solutions/flowjo
R Statistical Computing Software	The R Foundation	https://www.r-project.org/
Fiji (ImageJ) 1.53t	Fiji	https://imagej.net/
QuPath 0.6.0	QuPath	https://qupath.github.io/

## Data Availability

Fixed single-nucleus RNA sequencing data have been deposited in raw format at GEO: GSE305547. Previously published bulk RNA sequencing data of murine HFSCs and single-cell RNA sequencing data of murine skin and SDLN Tregs can be found at GEO: GSE76102, GSE175746, and GSE136160. Additionally, we downloaded healthy human skin single-cell RNA sequencing data from github.com/GreenleafLab/scScalpChromatin. This paper does not report any new code. All data reported in this paper will be shared by the lead contact upon request. Any additional information required to reanalyze the data reported in this paper is available from the lead contact upon request.

## Highlights

- Infundibular and isthmic hair follicle keratinocytes are enriched for Cxcl12
- Skin Tregs express Cxcr4, which promotes accumulation to hair follicle epithelium
- Glucocorticoid receptor expression by skin Tregs maintains Cxcr4
- The Cxcr4+ skin Treg/Cxcl12+ keratinocyte axis promotes optimal hair growth
