# Supplementary material for: Regulatory T cells in skin utilize the Cxcr4-Cxcl12 axis to promote hair follicle regeneration
Source: Cell Rep. Author manuscript; Available in PMC 2026 Feb 4. (PMC12871648; doi:10.1016/j.celrep.2025.116467)
Supplement: 1 [file NIHMS2125809-supplement-1.pdf]

Cell Reports, Volume *44*

Supplemental information

Regulatory T cells in skin utilize

the Cxcr4-Cxcl12 axis

to promote hair follicle regeneration

Jarish N. Cohen, Gayatri Kolluri, Sean Clancy, Victoire Gouirand, Courtney E. Macon, Lokesh A. Kalekar, and Michael D. Rosenblum

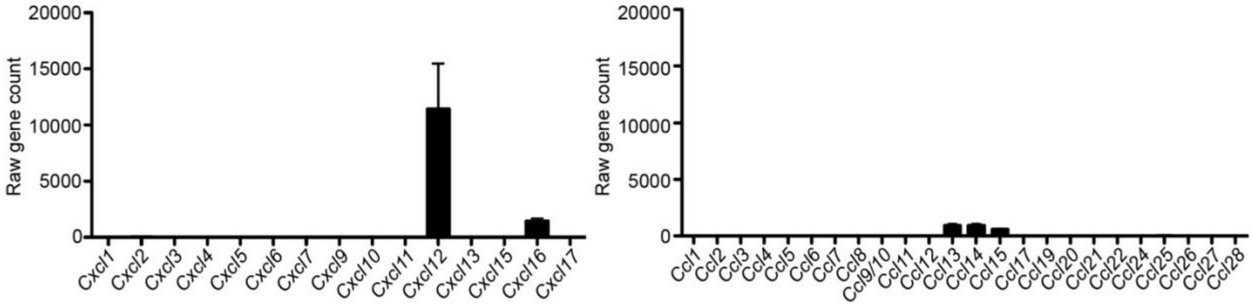

Figure S1. Chemokine gene expression by murine bulge hair follicle stem cells. Related to Figure 1. Raw gene counts of chemokine mRNA expression from sort-purified bulge hair follicle stem cells (gated on live CD45- Sca-1- EpCAM^{int} CD34+ cells). Data are representative of one experiment using triplicate samples.

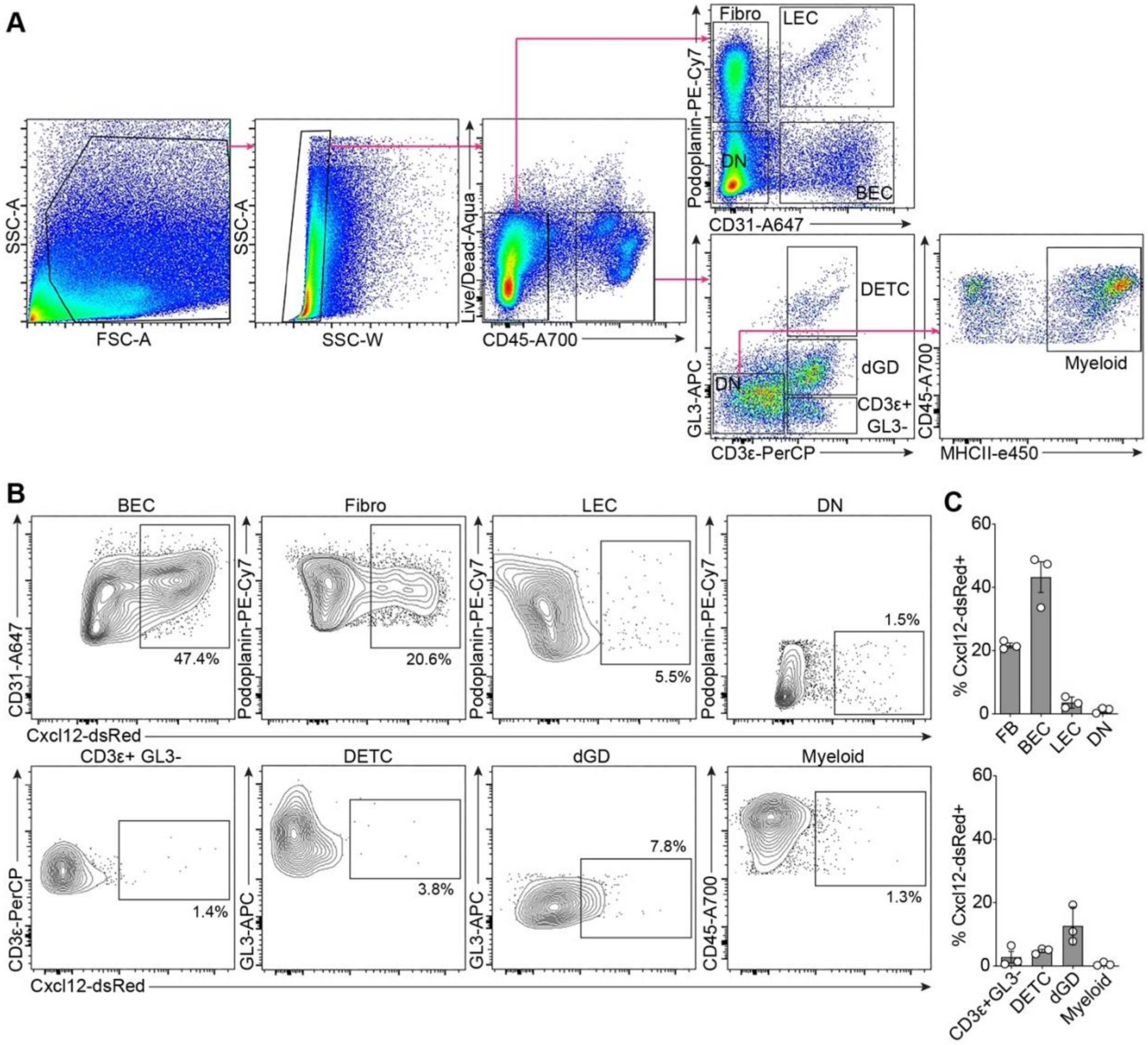

Figure S2. Characterization of Cxcl12 expression in cutaneous immune and stromal subsets. Related to Figure 1.

A. Flow cytometry gating strategy for cutaneous innate lymphoid and myeloid subsets (DETCs: dendritic epidermal T cells, dGDs: dermal γ/δ T cells, DN: double negative, BEC: blood endothelial cells; Fibro, FB: fibroblasts; LEC: lymphatic endothelial cells). **B**, **C**. Representative flow cytometry plots (**B**) and quantification (**C**) of %Cxcl12-dsRed+ cells in each of the indicated stromal and immune subsets. Data are representative of two independent experiments with similar results. Results are shown as individual data points and mean +/- s.e.m.

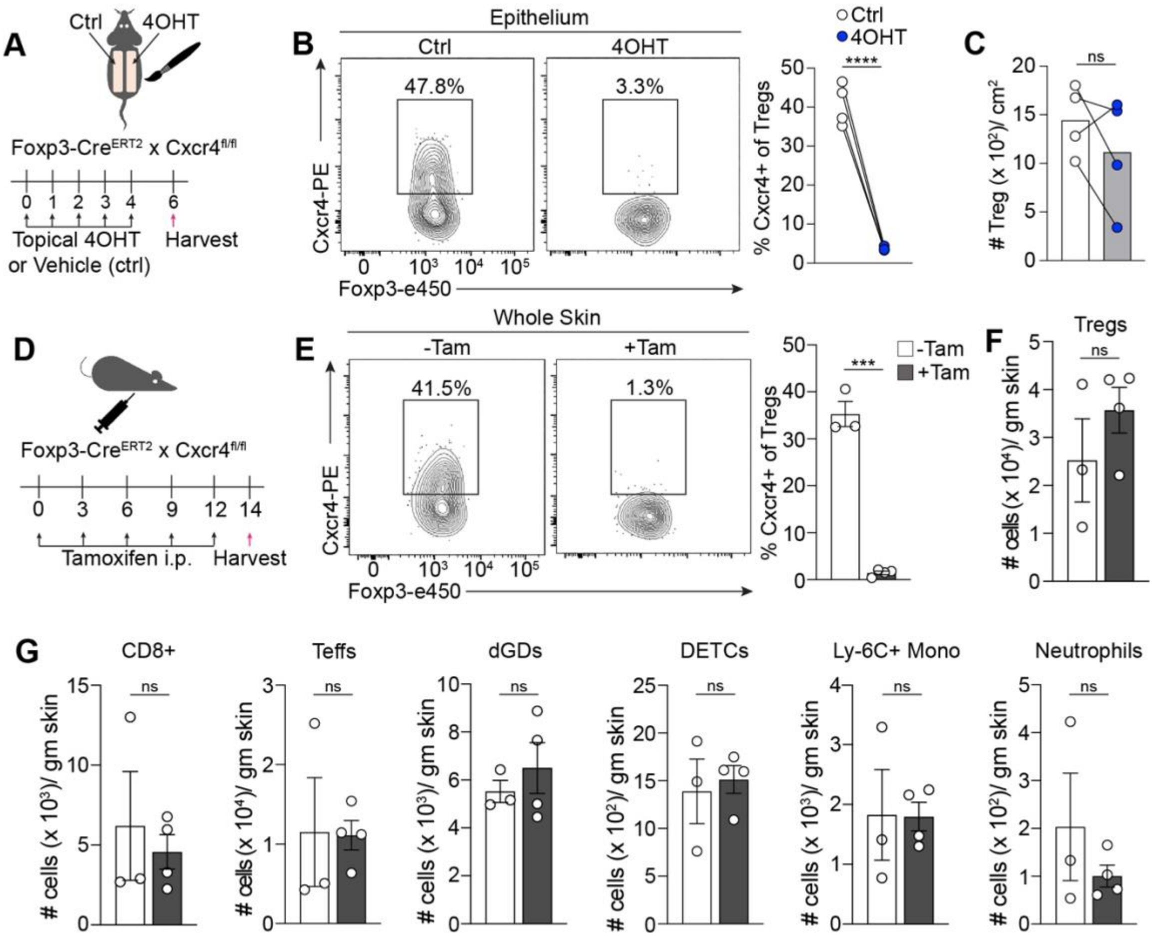

Figure S3. Cxcr4 expression on Tregs does not influence cutaneous immune homeostasis. Related to Figure 3.

A. Experimental schematic in which Foxp3-Cre^{ERT2} x Cxcr4$^{fl/fl}$ (Foxp3$^{\Delta iCxcr4}$) mice were treated for 5 consecutive days with 4OHT on one side of shaved back skin and vehicle control (acetone) on the contralateral side, and skin epithelium was enzymatically separated and analyzed 2 days later. **B**. Representative flow cytometry plots and quantification of % Cxcr4+ Tregs in skin epithelium. **C**. Quantification of the number of Tregs in skin epithelium. **D**. Experimental schematic in which Foxp3$^{\Delta iCxcr4}$ mice were treated every three days with Tamoxifen intraperitoneally (i.p.) for two weeks, and skin was harvested thereafter. **E**. Representative flow cytometry plots and quantification of % Cxcr4+ Tregs in whole skin. **F**. Quantification of the number of Tregs in whole skin. **G**. Quantification of the indicated lymphocyte and myeloid subsets in whole skin. Data are representative of two independent experiments with similar results. Results are shown as paired data points (**B**, **C**), and as individual data points and mean +/- s.e.m (**E**-**G**). Statistics are calculated by paired (**B**, **C**) and unpaired (**E**-**G**) two-tailed Student's *t*-test. ns = not significant.

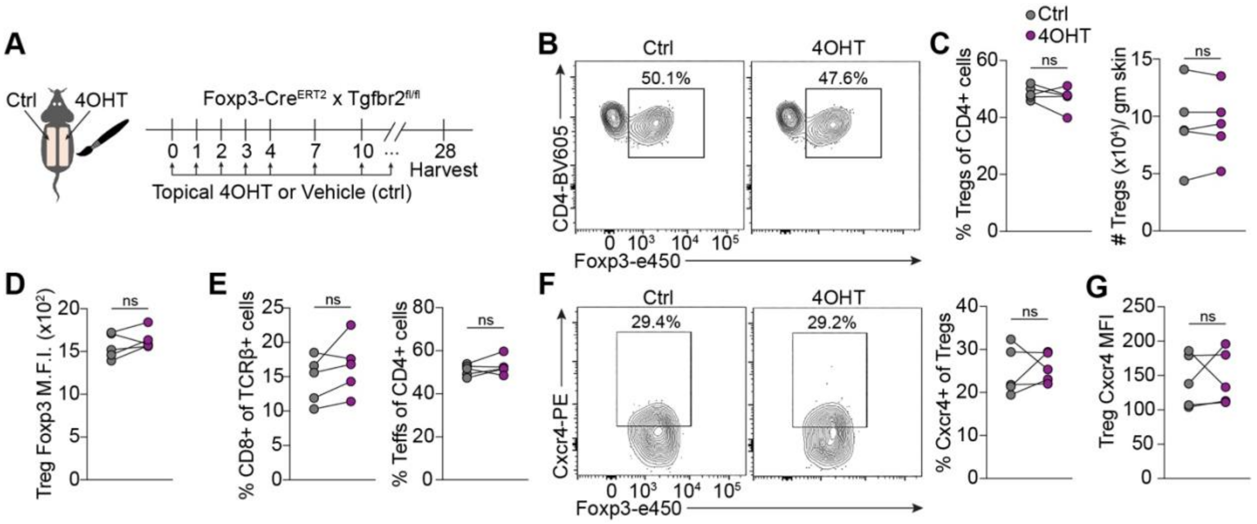

Figure S4. Tgfβ signaling on skin Tregs does not influence their accumulation or promote Cxcr4 expression. Related to Figure 4.
A. Experimental schematic in which Foxp3-Cre^{ERT2} x Tgfbr2$^{fl/fl}$ (Foxp3$^{\Delta iTgfbr2}$) mice were treated for 5 consecutive days with 4OHT on one side of shaved back skin and vehicle control (acetone) on the contralateral side, and every three days thereafter for 4 weeks. Skin was subsequently harvested. **B**. Representative flow cytometry plots of % skin Tregs. **C**. Quantification of the frequency and number of skin Tregs. **D**. Quantification of Foxp3 mean fluorescence intensity (M.F.I.) on skin Tregs. **E**. Quantification of the frequency of CD8+ T cells ad CD4+ T effector (Teff) cells in skin. **F**. Representative flow cytometry plots and quantification of % Cxcr4+ skin Tregs. **G**. Quantification of Cxcr4 mean fluorescence intensity (M.F.I.) on skin Tregs. Data are representative of two independent experiments with similar results. Results are shown as paired data points. Statistics are calculated by paired two-tailed Student's *t*-test. ns = not significant.

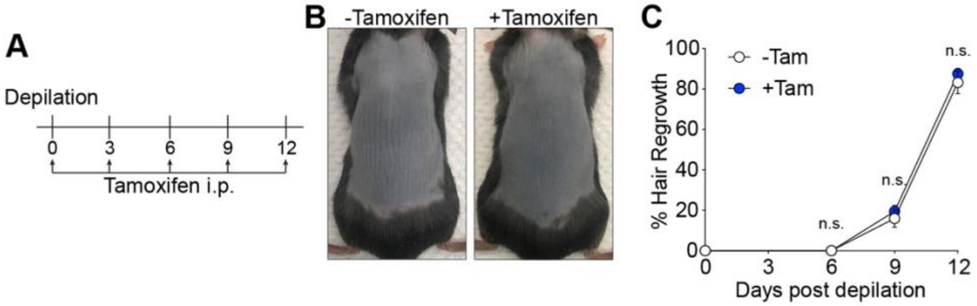

Figure S5. Systemic Tamoxifen treatment does not influence hair follicle regrowth following depilation. Related to Figure 5.
A. Experimental schematic in which Foxp3-Cre^{ERT2} mice were depilated and treated every 3 days with Tamoxifen injection intraperitoneally over 12 days. **B**. Representative gross photographs of mouse back skin nine days after chemical depilation. **C**. Quantification of hair regrowth at defined time points following chemical depilation. Data are representative of one experiment. Results are shown as mean +/- s.e.m. Statistics are calculated by unpaired two-tailed Student's *t*-test. n.s. = not significant.

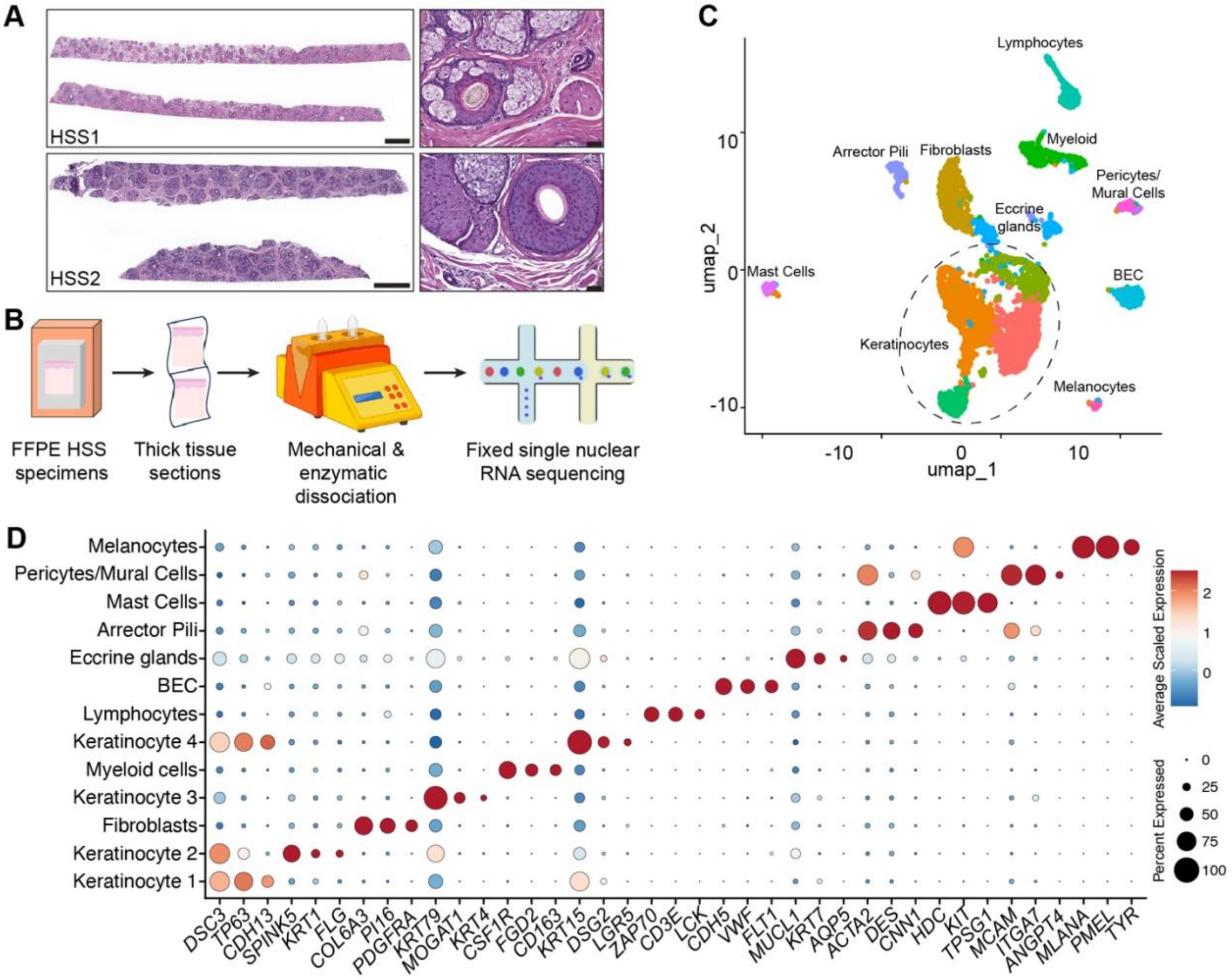

Figure S6. Fixed single nuclear RNA sequencing from archived healthy scalp skin. Related to Figure 6.

A. Low and medium-power photomicrographs of two hematoxylin and eosin (H&E)-stained tissue sections from two archived healthy scalp skin (HSS) specimens (bar = 2mm (left panels), bar = 50 µm (right panels)). **B**. Schematic of fixed single nuclear RNA sequencing method from archived formalin-fixed paraffin-embedded (FFPE) specimens. FFPE tissue blocks were retrieved and 40 µm thick tissue sections were cut as scrolls. Tissue scrolls underwent concurrent enzymatic digestion and mechanical dissociation. The resultant tissue suspensions were filtered and underwent droplet and capture-based single nuclear RNA sequencing (snFFPEseq). **C**. Unsupervised clustering of combined snFFPEseq data from HSS1 and HSS2, which identified many of the major constituents in human scalp skin (BEC: blood endothelial cells). **D**. DotPlot displaying differentially expressed genes for each cluster. Data are shown as combined results from two independent experiments. (**B**) was created with Biorender.

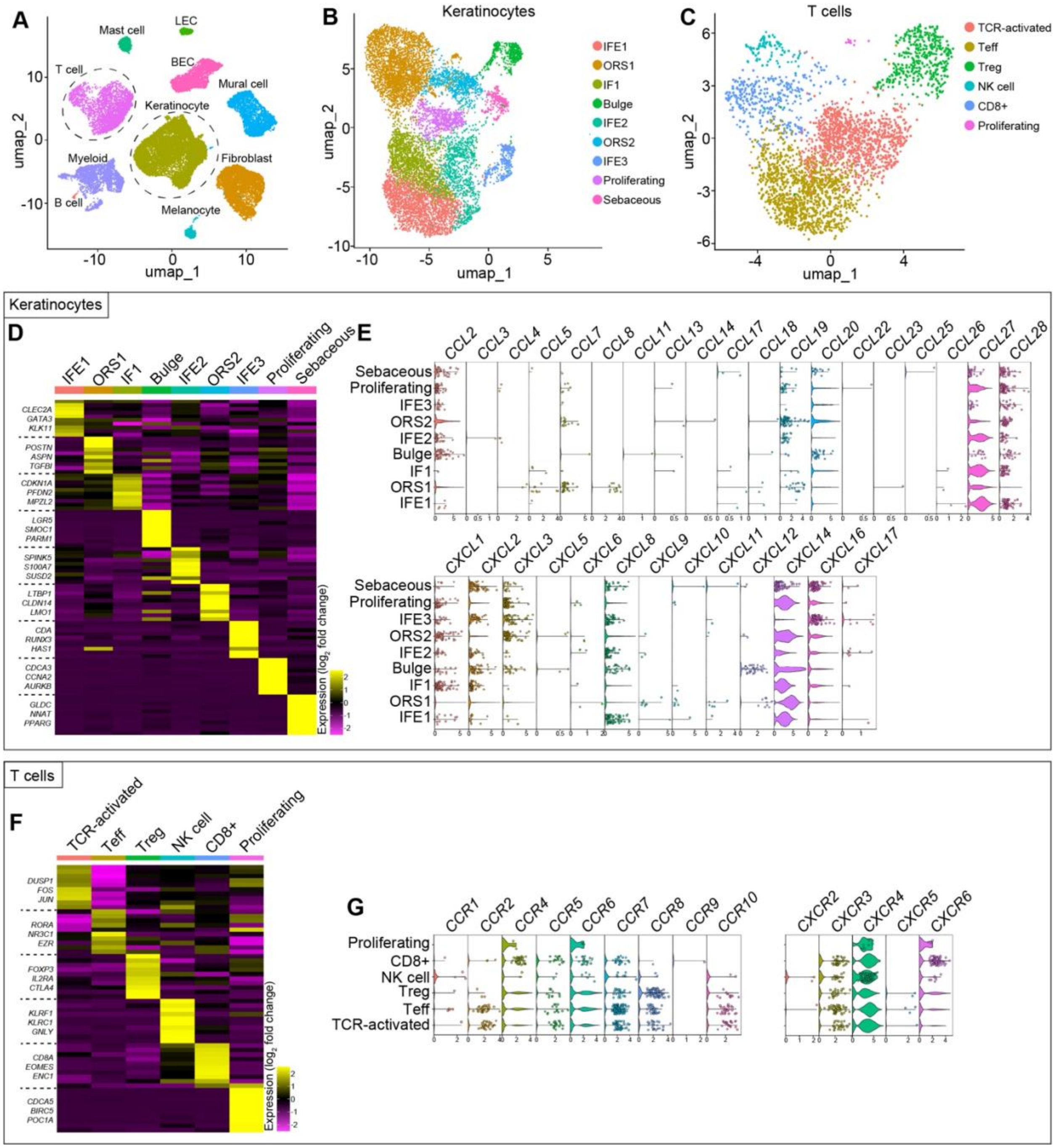

Figure S7. Analysis of published human scalp skin scRNAseq data confirms enrichment of cognate CXCR4 chemokines in keratinocytes of the upper hair follicle. Related to Figure 6.
A-C. Unsupervised clustering of scRNAseq data from ten healthy human skin specimens from Ober-Reynolds et al. [S1] showing total cellular constituents, keratinocytes (**B**) and lymphocytes (**C**). **D**, **F**. Heatmaps of representative differentially expressed genes of distinct clusters of keratinocytes (**D**) and lymphocytes (**F**). Violin plots of chemokine genes expressed by keratinocytes (**E**) and chemokine receptors expressed by lymphocytes (**G**). LEC: lymphatic endothelial cells, BEC: blood endothelial cells, IFE: interfollicular epidermis, IF: infundibulum, ORS: outer root sheath.

Table S1: Human scalp skin demographics.

Cohen et al.			
**Sample_ID**	**age**	**sex**	**preservation**
HSS1	72	F	FFPE
HSS2	53	M	FFPE
Ober-Reynolds et al.			
**Sample_ID**	**age**	**sex**	**preservation**
C_PB1	30	M	fresh
C_PB2	20	F	fresh
C_PB3	25	F	fresh
C_SD1	45	F	fresh
C_SD2	45	F	fresh
C_SD3	65	F	fresh
C_SD4	75	M	frozen
C_SD5	80	M	frozen
C_SD6	65	F	frozen
C_SD7	65	M	frozen

METHODS S1: Methods relating to supplementary material only

Bulk RNA sequencing of murine HFSCs and single cell RNA sequencing of murine Tregs

Murine HFSCs were sort-purified, underwent mRNA extraction, sequenced, and data analyzed as in [S2]. Murine skin Tregs were isolated from four Foxp3-Cre$^{ERT2\text{-}GFP}$ adult female mice. SDLN Tregs were prepared by harvesting pooled inguinal, brachial, and axillary LNs from two Foxp3-GFP adult female mice. Samples underwent single cell RNA sequencing as described in [S3-S5].

Single cell RNA sequencing analysis of a previously published healthy scalp skin dataset

Processed healthy scalp skin scRNA-seq objects were obtained from github.com/GreenleafLab/scScalpChromatin [S1]. Downstream data analysis, including clustering, visualizations, and exploratory analyses, were performed in Seurat R package 5.1.0 using the processed "scalp", "Keratinocytes", and "Lymphoid" objects provided by the authors. After removing alopecia areata samples, markers for each cluster were identified with the Seurat *FindAllMarkers* function and *FineClust* annotations from the authors (Wilcoxon rank sum test, min.pct = 0.01, only.pos = True, thresh.use = 0.25), and cells were annotated according to known expression of DEGs. Log-normalized gene expression data were used for visualizations with UMAP plots (*FeaturePlot*). Keratinocyte and lymphocyte clusters were separately subclustered, normalized and scaled. The Eccrine subcluster (expression of *KRT7*, *KRT19*, *DCD* genes) was removed from the "Keratinocytes" object. For violin plots (*VlnPlot*) we added points for populations with total counts <100 and points with expression values >0. Heatmaps were generated on the RNA assay using the PseudobulkExpression function using the top 10 marker genes arranged by adjusted p-values and descending $\log_2$FC.

Supplementary References

1. Ober-Reynolds, B., Wang, C., Ko, J.M., Rios, E.J., Aasi, S.Z., Davis, M.M., Oro, A.E., and Greenleaf, W.J. (2023). Integrated single-cell chromatin and transcriptomic analyses of human scalp identify gene-regulatory programs and critical cell types for hair and skin diseases. Nat Genet *55*, 1288–1300. https://doi.org/10.1038/s41588-023-01445-4.

2. Ali, N., Zirak, B., Rodriguez, R.S., Pauli, M.L., Truong, H.-A., Lai, K., Ahn, R., Corbin, K., Lowe, M.M., Scharschmidt, T.C., et al. (2017). Regulatory T Cells in Skin Facilitate Epithelial Stem Cell Differentiation. Cell *169*, 1119-1129.e11. https://doi.org/10.1016/j.cell.2017.05.002.

3. Cohen, J.N., Gouirand, V., Macon, C.E., Lowe, M.M., Boothby, I.C., Moreau, J.M., Gratz, I.K., Stoecklinger, A., Weaver, C.T., Sharpe, A.H., et al. (2024). Regulatory T cells in skin mediate immune privilege of the hair follicle stem cell niche. Sci Immunol *9*, eadh0152. https://doi.org/10.1126/sciimmunol.adh0152.

4. Kalekar, L.A., Cohen, J.N., Prevel, N., Sandoval, P.M., Mathur, A.N., Moreau, J.M., Lowe, M.M., Nosbaum, A., Wolters, P.J., Haemel, A., et al. (2019). Regulatory T cells in skin are uniquely poised to suppress profibrotic immune responses. Sci Immunol *4*. https://doi.org/10.1126/sciimmunol.aaw2910.

5. Moreau, J.M., Dhariwala, M.O., Gouirand, V., Boda, D.P., Boothby, I.C., Lowe, M.M., Cohen, J.N., Macon, C.E., Leech, J.M., Kalekar, L.A., et al. (2021). Regulatory T cells promote innate inflammation after skin barrier breach via TGF-β activation. Sci Immunol *6*, eabg2329. https://doi.org/10.1126/sciimmunol.abg2329.
